# Do interventions for mood improve inflammatory biomarkers in inflammatory bowel disease?: a systematic review and meta-analysis

**DOI:** 10.1016/j.ebiom.2023.104910

**Published:** 2024-01-24

**Authors:** Natasha Seaton, Joanna Hudson, Sophie Harding, Sam Norton, Valeria Mondelli, Annie S.K. Jones, Rona Moss-Morris

**Affiliations:** Institute of Psychology Psychiatry and Neuroscience, King’s College London, UK

**Keywords:** Inflammatory Bowel Disease, Depression, Anxiety, Psychological intervention, Meta-analysis, Psychoneuroimmunology

## Abstract

**Background:**

Psychoneuroimmunological mechanisms and the gut-brain axis appear relevant to disease activity and progression in Inflammatory Bowel Disease (IBD). A recent review showed no effect of psychological therapies on self-reported disease activity in IBD. This meta-analysis aims to establish whether interventions targeting mood outcomes (e.g., depression, anxiety and stress) impact inflammation levels in IBD and possible moderators of these effects.

**Methods:**

The Preferred Reporting Items for Systematic Reviews and Meta-analyses (PRISMA) guidelines were followed. We searched five electronic databases and included randomised controlled trials where interventions targeted mood and assessed inflammatory outcomes pre- and post-intervention in adults with IBD. Independent reviewers screened studies, extracted data, and assessed methodological quality. Data were pooled to estimate standardised mean differences (SMDs) with 95% Confidence Intervals (CIs). A random-effects robust variance estimation accounted for studies measuring multiple biomarkers. Intervention type, mood as a primary or secondary outcome, effect on mood outcomes and IBD subtype were investigated as treatment effect moderators. Where there were sufficient biomarkers, individual meta-analyses were run (Pre-registration PROSPERO: CRD42023389401).

**Findings:**

28 RCTs involving 1789 participants met inclusion criteria. Interventions demonstrated small, statistically significant effects on biomarkers (−0.35, 95% CI: −0.48, −0.22, p < 0.001) and medium effects on mood outcomes (−0.50, 95% CI: −0.73, −0.27, p < 0.001), without evidence of substantive heterogeneity or publication bias. Individual analyses showed small effects for improved faecal calprotectin (−0.19, 95% CI: −0.34, −0.03, p = 0.018) and C-Reactive Protein (−0.29, 95% CI: −0.47, −0.10, p = 0.002). Effect sizes were larger for psychological therapy interventions (compared with exercise or antidepressants) and when there was an effect (SMD ≥0.2) on mood.

**Interpretation:**

Treatments which address mood outcomes have beneficial effects on generic inflammation as well as disease-specific biomarkers (faecal calprotectin and C-Reactive Protein). Psychological interventions and interventions with larger treatment effects on mood accentuated the effect on biomarkers. More research is required to understand the biological or behavioural mechanisms underlying this effect.

**Funding:**

The 10.13039/501100000265Medical Research Council and the 10.13039/100019418National Institute for Health and Care Research (NIHR) Maudsley Biomedical Research Centre.

**Research in context**

**Evidence before this study**

There is a growing body of evidence that psychosocial factors are critical considerations within medicine and immunology, particularly for inflammatory autoimmune conditions such as Inflammatory Bowel Disease (IBD). Although mood disorders, such as depression, anxiety and distress, have been shown to impact symptom severity and progression in IBD, the mechanistic pathways are not well understood. Previous reviews and meta-analyses have attempted to establish whether psychological treatments can improve self-reported disease activity; however, none have synthesised the effects of interventions designed to treat mood on inflammatory biomarkers. We searched MEDLINE, EMBASE, PsycINFO, Global Health and Web of Science from inception to 24 October 2023. We found 28 original articles that were interventions of adults living with IBD that included mood as an outcome as well as an inflammatory biomarker outcome at pre- and post-intervention.

**Added value of this study**

This systematic review and meta-analysis of randomised controlled trials demonstrates that interventions for mood result in significant reductions in inflammatory biomarkers. This includes both biomarkers that are specific indicators of IBD disease activity (C-Reactive Protein: −0.29 [95% CI: −0.47, −0.10]; Faecal Calprotectin: −0.19 [95% CI: −0.34, −0.03]), and generic markers of inflammation (composite analysis: −0.35 [95% CI: −0.48, −0.22]). Interventions had a greater effect on biomarkers if they had a larger positive effect on mood and if they were psychological therapy, suggesting that improvements in mood directly influence the immune system and inflammation through psychoneuroimmunological pathways. Although there was low study heterogeneity, potential bias was identified with inadequate reporting of results.

**Implications of all the available evidence**

Interventions for mood show considerable promise for the management of IBD in improving mental health, inflammation and disease outcomes. Integrated mental health support alongside pharmacological treatments may offer a more holistic approach to IBD care, potentially leading to reduced disease activity and healthcare costs. Our analysis suggests that the treatment of mood directly improves inflammation; however, more research is needed to understand exact mechanisms. Future research should prioritise interventions that are shown to be effective at improving mood in IBD, assess inflammation and disease activity at multiple timepoints and include potential mediator variables relating to illness self-management.

## Introduction

Inflammatory Bowel Disease (IBD), comprising Crohn's disease (CD) and ulcerative colitis (UC), is a chronic autoimmune inflammatory condition of the gastrointestinal tract,^1^^,^^2^ characterised by periods of remission and activity, called relapse or flares.^3^ Physical symptoms are usually managed through immunosuppressive medications and surgery. However, alongside the physical symptoms, IBD poses substantial psychosocial challenges, with rates of depression as high as 25.3% and anxiety as high as 32.1%, rising to 38.9% and 57.6%, respectively, with active disease.^4^ This effect is often attributed to increased symptom burden during active periods.^5^, ^6^, ^7^

A recent meta-analysis demonstrated that depression and anxiety predict poorer future clinical outcomes in IBD, including development of flares, hospitalisation, emergency department attendance, therapy escalation, and surgery.^8^ Moreover, in 718 IBD participants, having a common mental disorder worsened IBD prognosis, independently of baseline disease activity.^9^ Given the relationship between disease activity and mood, addressing mental health concerns should be a priority for IBD treatment.^9^

Meta-analyses of randomised controlled trials (RCTs) across different medical conditions demonstrated that psychosocial interventions reduced inflammation and levels of proinflammatory cytokines by 18–48%, when compared with controls.^10^^,^^11^ Exact mechanisms are not fully understood, but interventions which reduce negative psychological factors appear to subsequently impact immune system function and inflammation, partly through activation of the parasympathetic nervous system.^12^, ^13^, ^14^ This may be relevant for IBD, as psychological and behavioural factors are likely to impact intestinal inflammation and disease activity through the gut-brain axis.^15^ A recent meta-analysis of RCTs trialling psychological therapies specifically in IBD found no improvement in disease activity defined as remission status, relapse activity, or use of a self-report questionnaire.^16^ The psychological interventions included in this review yielded small effects for anxiety, depression, and stress (Cohen’s *d* = −0.23, −0.26, −0.22, respectively). Given that hypothesised changes in mood, should, via the gut-brain axis, cause inflammatory improvements, studies demonstrating only small effects on mood may be less likely to improve disease activity or enhance immune function. Moreover, the review was restricted to quiescent IBD, which may have limited its ability to detect significant effects on disease activity.

The current review aims to address the remaining gaps in the literature. The primary outcomes are objective markers of inflammation so that firstly, the mechanisms of action through which psychological interventions might improve disease activity will be explored and secondly, we will avoid using self-report indices which often correlate poorly with objective disease indicators.^17^ Moreover, a broader range of interventions used to target mood and mental health outcomes, such as exercise interventions, will be included. Consequently, this will enable moderator analyses, including size of treatment effects on mood and intervention type, which may elucidate potential mechanisms. Moreover, whether mood is a primary or secondary outcome in the trial will be a moderator to distinguish between interventions where mood is the primary target, or when mood improvements are expected because another concern (e.g., fatigue or pain) is being addressed.

This meta-analysis aims to further understanding of the complex interplay between mood and inflammation by answering the following research questions:

(1) Do interventions that aim to treat mood as a primary or secondary outcome significantly improve levels of inflammatory biomarkers in individuals with IBD?
(2) Does the size of the intervention’s effect on mood, whether mood was a primary or secondary outcome, intervention type, and disease subtype moderate the effect of interventions for mood on inflammatory markers?

## Methods

The review protocol was registered prospectively (PROSPERO CRD42023389401). The research objectives were refined, given the data available in the studies suitable for inclusion. Findings were reported in line with The Preferred Reporting Items for Systematic Reviews and Meta-analyses (PRISMA) guidelines^18^ (Supplementary Table S1).

### Eligibility criteria and selection process

Two reviewers (NS and SH) independently screened studies against PICOS (Population, Intervention, Comparison, Outcome, Study design) criteria (Table 1), as a gold standard screening methodology.^19^ Disagreements were resolved by consensus discussion. Eligible studies were RCTs in adults with IBD that trialled interventions that included mood (depression, anxiety, stress, distress, or poor emotional wellbeing) as a primary or secondary outcome. All studies had to include an inflammatory biomarker, measured pre- and post-intervention. Demographics and mood scores were collected, if available.

**Table 1:** PICOS inclusion/exclusion criteria.

	Inclusion criteria	Exclusion criteria
Population	• Adults (≥18 years) with IBD (radiologically, histologically or endoscopically confirmed or self-report diagnoses) • Active and inactive disease	• No IBD diagnosis • Animal studies
Intervention	• Psychosocial (including anti-depressants) and/or behavioural intervention studies • Measure of mood as primary/secondary outcome	• Drug interventions that are not anti-depressants • Mood not a primary or secondary outcome
Comparator	• Any control group (usual care, waitlist control, placebo control or active control)	• No comparator
Outcome	• Inflammatory biomarkers (including, but not limited to, faecal calprotectin, C-Reactive Protein, endoscopic inflammation, pro-inflammatory cytokine activity)	• Self-report clinical indices • Biomarkers that do not measure inflammation
Study design	• RCT's or quasi-RCT's	• Non-randomised studies

IBD: inflammatory bowel disease; RCT: randomised controlled trial.

### Search strategy

A systematic, comprehensive literature search was conducted in MEDLINE, EMBASE, PsycINFO, Global Health (through Ovid, 2022) and Web of Science (through Clarivate 2022), from 1947 to October 2023. No language limits were imposed, and foreign language articles were translated. Additionally, citations within relevant articles were screened to identify articles not retrieved by the search. Where protocols or conference abstracts were identified, full texts were requested from authors.

The search strategy included medical subject headings (MeSH) and multi-purpose terms (e.g., title, abstract, keywords). There were 3 categories, pertaining to 1) IBD; 2) mood; and 3) inflammation/disease activity. Appropriate Boolean operators were used (Supplementary Table S2).

### Data extraction

References were exported to EndNote v20. Data were extracted using a Microsoft Excel Spreadsheet (Microsoft Office 365, version 2023). For all inflammatory and mood outcomes the sample size, mean and standard deviation for each group were extracted. If these data were not available, categorical data and/or mean differences were extracted. Where studies used more than one outcome for either mood or inflammatory biomarkers, all outcomes were extracted. Authors were contacted if data were missing or unclear. If both dichotomous and continuous data were presented, continuous data was prioritised. Two reviewers independently extracted data from primary sources (NS and AJ). One author (NS) used the Template of Intervention Description and Replication (TIDieR) guidance^20^ to extract intervention details from main publications, Supplementary Material and study protocols.

### Moderator coding

The key moderators, selected a-priori, were 1) intervention type; 2) whether mood outcomes were listed as primary or secondary outcomes; and 3) the type of IBD included in the study. A further moderator, 4) intervention effect size on mood, was added during data extraction based on the presentation of outcomes in eligible articles. Categorical moderators were dummy coded. Intervention type was anticipated to fall into three categories: exercise, psychotropic/antidepressant (defined as the administration of antidepressants for mood) and psychological therapy (e.g., Cognitive Behavioural Therapy (CBT), Acceptance and Commitment Therapy (ACT), Mindfulness Based Stress Reduction (MBSR), or other psychological intervention techniques beyond usual care or information provision). Due to the limited range of eligible studies, we were unable to assess differences between types of therapies within these categories. Studies were only coded as having mood as a primary outcome if this was explicitly stated: studies with no distinction were coded as secondary outcomes. For IBD-subtype, analyses that did not distinguish between diagnoses (e.g., Crohn’s Disease and Ulcerative Colitis) were excluded. Finally, interventions were considered to have an effect on mood if the effect size for mood improvements was ≥0.20, indicating at least a small effect size.^21^

### Risk of bias (RoB) assessment

The methodological RoB of each study was assessed independently by two reviewers (NS and JH/SH) according to Cochrane Handbook guidance^22^ and the revised tool for assessing RoB in RCTs.^23^ This includes bias arising from: the randomisation process (sequence allocation, baseline differences between groups); deviations from intended interventions (blinding of participants/outcome assessments, protocol deviations); missing outcome data; outcome measurement; and selection of reported results.

Publication bias was assessed using Egger’s test and the precision-effect-test and precision-effect estimate with standard error approach (PET-PEESE)^24^ to establish if studies were more likely to be reported if they had significant results and to calculate an adjusted estimate of the effect.

### Statistical methods

Data were analysed using STATA v17.0 from 05 July, 2023 to 07 November, 2023 (see Supplementary Material S1 for syntax; data available in online repository: https://data.mendeley.com/datasets/svtmy3274p/1). The primary a-priori effect size outcome of interest was the standardised mean difference (SMD) between the psychosocial intervention and control groups post-intervention. The *metaff* stata command was used to calculate standardised mean differences (SMDs) and 95% confidence intervals (95% CIs) for each effect size, including transforming effects for dichotomous outcomes into SMDs, allowing inclusion of both continuous and dichotomous outcomes in the same analysis. Effect sizes were interpreted using Cohen’s conventions, where an SMD of 0.2 represents a small effect, 0.5 a medium effect, and 0.8 a large effect.^21^ For studies that included multiple active comparator trial arms relative to the control group, we divided the control group sample size by the number of active arms to prevent double counting of participants, and consequent spurious precision.^22^

An overall SMD of the effect across all biomarkers was estimated. Many studies reported more than one type of immunologic outcome.^25^ Therefore, a robust variance meta-regression was used to provide a composite analysis of all biomarkers, accounting for the dependence between biomarkers.^26^ This enabled accurate estimation of effect size weights, standard errors and 95% CIs for multiple outcomes within studies. The robust variance meta-regression methodology was also deemed appropriate for assessing whether the interventions improved mood, as there were similar issues with studies measuring multiple measures of mood.

These analyses were conducted using the STATA *robumeta* command. A recommended value of ρ = 0.80^26^ was used to account for the dependency between immunological outcomes. Selecting different values of ρ between 0.1 and 0.9 did not impact the significance or precision of the overall effect. Heterogeneity was assessed using τ^2^, representing between-study variance. However, as heterogeneity is incidental to the robust variance meta-regression analysis, these values are reported without test statistics or significance levels.^26^ Sensitivity analyses conducted were 1) excluding influential points (defined as DFBETA value > $\frac{2}{\sqrt{n}}$) and 2) excluding results from psychotropic/antidepressant interventions. Egger’s tests, clustered by study, tested publication bias. We anticipated there would be insufficient studies to include all moderators into one meta-regression model. Therefore, separate robust variance meta-regressions were run for each moderator.

In line with Cochrane guidance, individual meta-analyses were run for biomarkers recorded in at least 10 studies. The *admetan* command, with a dersimonian–laird random-effects model, was used. I^2^ estimated statistical heterogeneity, with values ≥40% representing moderate heterogeneity and ≥75% representing high heterogeneity.^22^ Funnel plots graphically indicated asymmetry of the data that might have demonstrated possible publication bias. Additionally, a leave-one-out meta-analysis^27^ was conducted to determine the influence of omitting each study from the analysis on the pooled effect, with the magnitude of influence for each study summarised using the DFBETA statistic calculated for the specific biomarker. As with above, sensitivity analyses were conducted excluding influential points (defined as DFBETA value > $\frac{2}{\sqrt{n}}$).

Negative effect sizes indicate that the intervention group had either reduced inflammatory activity or increased anti-inflammatory activity relative to the controls, with positive effect sizes signalling the opposite. Similarly, negative effect sizes for mood indicated reduced depression/anxiety/stress relative to controls, with positive effect sizes signalling improved quality of life/wellbeing.

### Role of funding source

The funders were not involved in data collection, analysis, interpretation, design, nor in the decision to submit for publication.

## Results

### Study selection

The search identified 21,101 articles, which resulted in 15,631 original references after removing duplicates. Screening of titles removed 15,489 studies as they were clearly ineligible based on one or more inclusion criterion. 142 studies were identified for full text screening, with 106 not meeting inclusion criteria. This left 36 eligible studies, whose reference lists were searched, yielding no additional eligible studies. Of these 9 reported full data and 27 were contacted to provide complete data. Of these, 9 responded with full data, 11 did not provide complete data but had some usable data, 3 studies were ongoing and 5 did not respond in the absence of usable data (Citations in Supplementary Table S3).

**Fig. 1:**
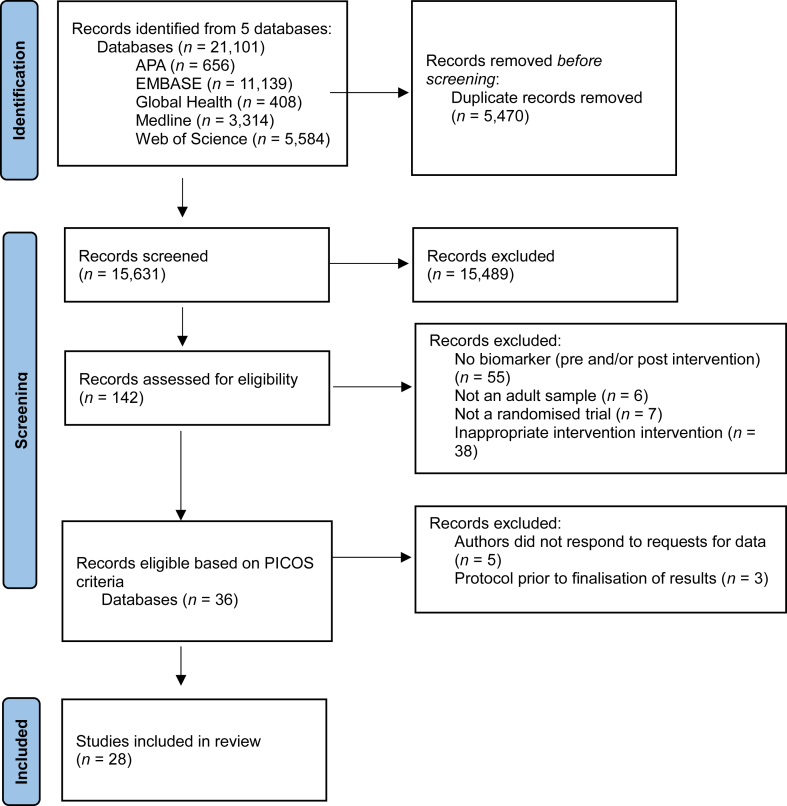
Study selection flowchart.

This left 28 unique RCTs^28^, ^29^, ^30^, ^31^, ^32^, ^33^, ^34^, ^35^, ^36^, ^37^, ^38^, ^39^, ^40^, ^41^, ^42^, ^43^, ^44^, ^45^, ^46^, ^47^, ^48^, ^49^, ^50^, ^51^, ^52^, ^53^, ^54^, ^55^ of 1789 participants (Fig. 1). Intercoder agreement was 99.96% (Cohen’s κ = 0.91). Two studies trialled two active interventions against a control group^48^^,^^51^ and 1 study trialling three active interventions against a control group,^42^ resulting in 32 pairwise comparisons. The number of inflammatory biomarkers measured in studies ranged from 1 to 21 (mean = 3.61, SD = 3.67), giving rise to 116 treatment effect estimates of biomarkers. Detailed study characteristics are presented in Supplementary Table S4. 22 arms from 20 studies trialled psychological therapy,^28^^,^^31^, ^32^, ^33^, ^34^, ^35^, ^36^, ^37^, ^38^^,^^40^^,^^41^^,^^43^^,^^44^^,^^46^^,^^49^, ^50^, ^51^, ^52^^,^^54^^,^^55^ 3 studies trialled antidepressants^42^^,^^45^^,^^53^ and 6 arms from 5 studies trialled exercise interventions.^29^^,^^30^^,^^39^^,^^47^^,^^48^ Details of intervention format and content, based on TIDieR guidance, are reported in Supplementary Table S5.

### Overall effect size

**Fig. 2:**
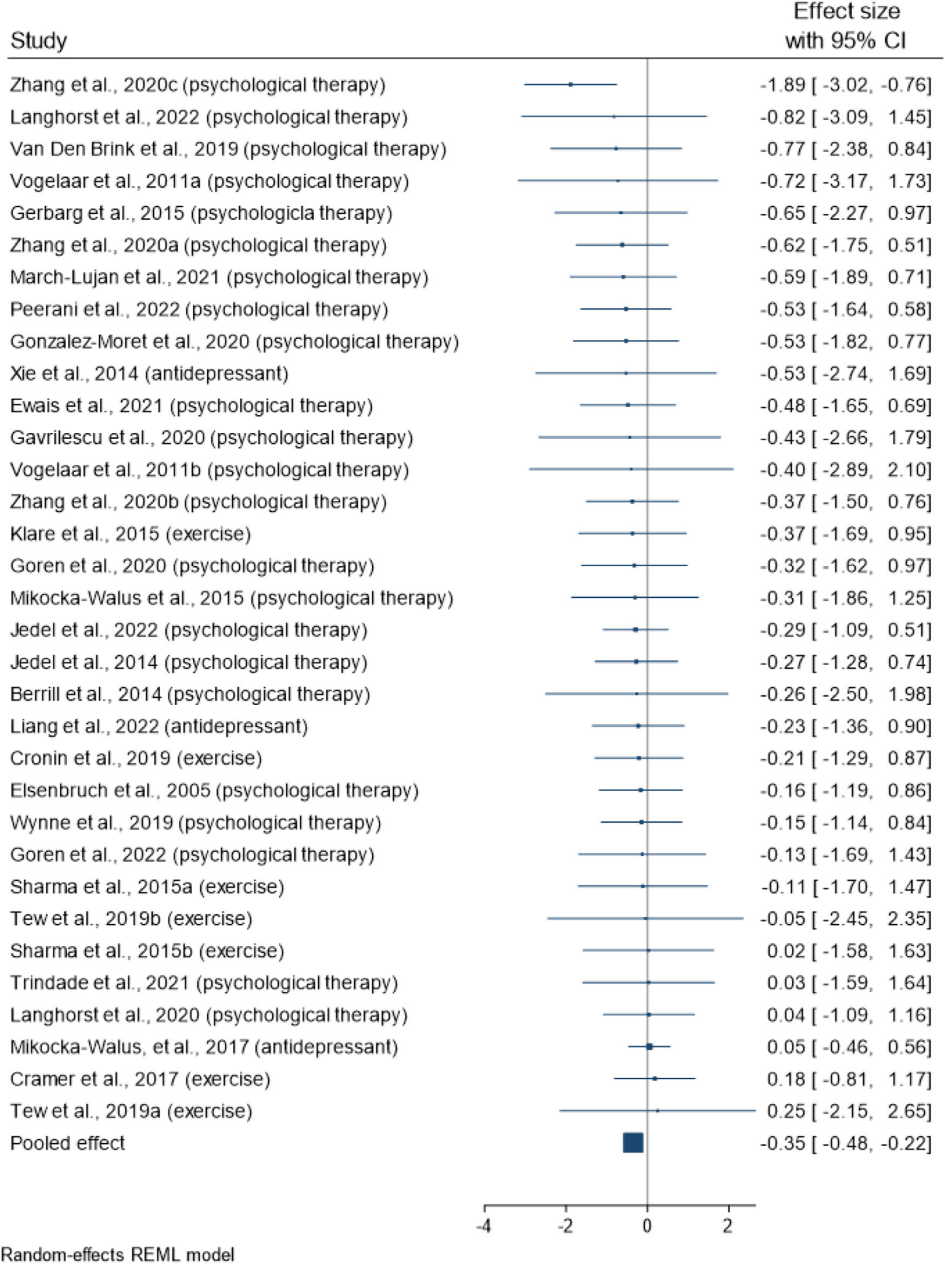
Forest plot showing study average effects of psychosocial/behavioural interventions on inflammatory biomarkers.

For the primary research question, the pooled effect from the robust variance meta-analysis of all biomarkers demonstrated that, interventions treating mood significantly reduced levels of inflammatory biomarkers compared with controls (SMD = −0.349, 95% CI: −0.48 to −0.22; z = −5.44, p < 0.001), corresponding to an 18% reduction in inflammatory biomarkers (Fig. 2). There was low heterogeneity between the effect sizes (τ^2^ < 0.001) and no evidence of publication bias (Egger’s test = −0.069, p = 0.833). Eleven biomarker effect sizes from three studies^31^^,^^45^^,^^54^ exceeded the DFBETA threshold. When these were excluded in a sensitivity analysis, interventions for mood continued to demonstrate statistically significant reduced levels of inflammation compared with controls (SMD = −0.302, 95% CI: −0.41 to −0.19, z = −5.67, p < 0.001), with low heterogeneity (τ^2^ < 0.001) and no evidence of publication bias (Egger’s test = 0.039, p = 0.953). Additionally, when the three antidepressant studies^42^^,^^45^^,^^53^ were excluded in a separate sensitivity analysis, the effect amongst the remaining 29 study arms (90 effect sizes) remained significant (SMD = −0.357, 95% CI: −0.48 to −0.23, z = −5.67, p < 0.001) with low heterogeneity (τ^2^ < 0.001) and no evidence of publication bias (Egger’s test = −1.13, p = 0.136). An additional sensitivity analysis showed that neither average age nor sex ratio of studies moderated the relationship (See Supplementary Figures S1 and S2 for scatter plots).

### Faecal calprotectin

**Fig. 3:**
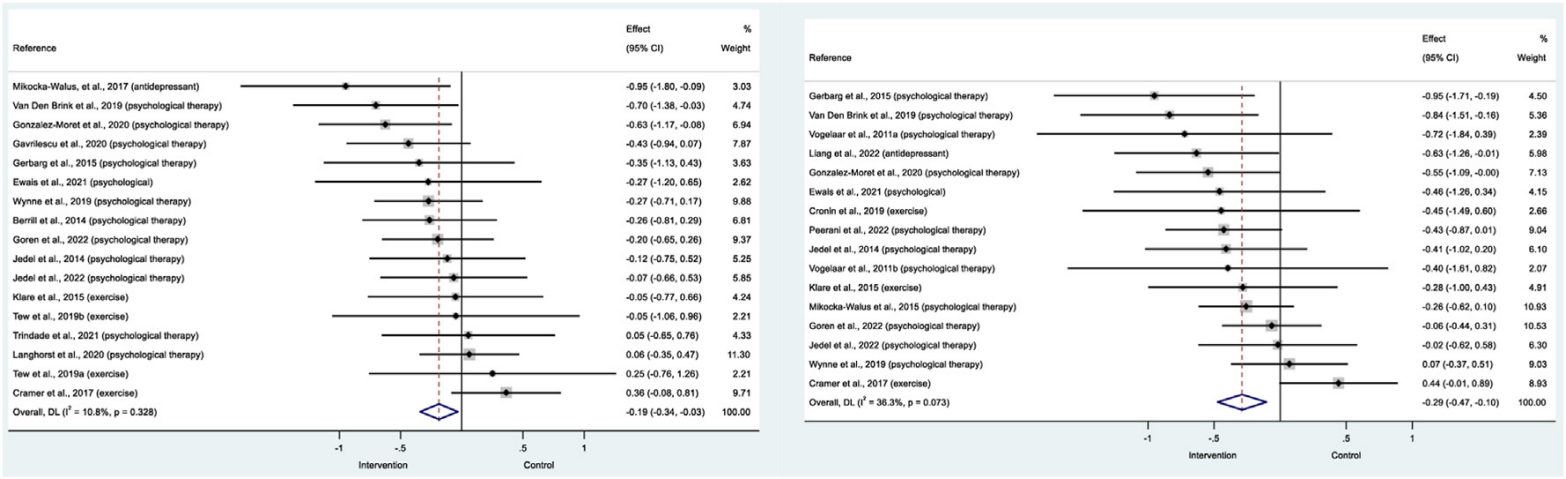
Forest plot showing effect of psychosocial/behavioural interventions on faecal calprotectin (left) and C-Reactive Protein (right).

Across the 17 study groups that investigated faecal calprotectin,^28^^,^^29^^,^^32^, ^33^, ^34^^,^^36^, ^37^, ^38^, ^39^^,^^41^^,^^45^^,^^48^, ^49^, ^50^^,^^52^^,^^55^ levels were significantly lower following interventions treating mood compared with controls (SMD = −0.186, 95% CI: −0.34 to −0.03; z = −2.38; p = 0.018; Fig. 3). When the effect size was back transformed using the pooled standard deviation, this corresponded to a reduction of 91 μg/g. There was no significant heterogeneity between the studies (I^2^ = 10.8%, p = 0.328), nor evidence of publication bias (Egger’s test = −0.876, p = 0.371; funnel plot shown in Supplementary Figure S3). Though non-significant, the PET analysis indicated potential bias from small sample effects (PET = 0.074, p = 0.811). The leave-one-out analysis showed that the removal of any study from the analysis did not substantively alter the effect size estimate (Supplementary Figure S4) and all comparisons had acceptable DFBETAs.

### CRP

CRP was assessed in 16 study groups,^29^^,^^30^^,^^32^^,^^34^^,^^36^, ^37^, ^38^, ^39^^,^^42^^,^^44^^,^^46^^,^^50^, ^51^, ^52^^,^^55^ demonstrating a small significant effect (SMD = −0.289, 95% CI: −0.47 to −0.10, z = −3.06, p = 0.002; Fig. 3). When the effect size was back transformed using the pooled standard deviation, this corresponded to a reduction of 2.44 mg/dL. There was significant heterogeneity (I^2^ = 36.3%, p = 0.073) and evidence of publication bias (Egger’s = −1.815, p = 0.013; Supplementary Figure S5 for funnel plot). Although non-significant, the PET analysis indicated potential imprecision in the effect (PET = 0.241, p = 0.292). The leave-one-out analysis showed the effect size did not significantly change with the removal of any study (Supplementary Figure S6) from the analysis. One comparison^29^ exceeded the DFBETA threshold: when this study was excluded, an effect of −0.322 was observed (95% CIs: −0.47 to −0.18, z = −4.29, p < 0.001), with low heterogeneity (I^2^ = 0.0%, p = 0.494), minimal imprecision in the effect (PET = 0.087, p = 0.60) and some evidence of publication bias (Egger’s test = −1.493, p = 0.014).

### Moderator analyses

The study aimed to examine whether the overall association of interventions treating mood on biomarkers was moderated by 1) intervention effect on mood outcomes; 2) intervention type; 3) whether mood was a primary/secondary outcome and 4) the type of IBD included in the study (Table 2).

**Table 2:** Categorical moderators of the association between psychosocial interventions and effect on levels of inflammation.

Moderator	No. of study groups	Effect size estimate, SMD (95% CI)	χ^2^ or z value (df)	p value
**Effect on mood**^a^			χ^2^ = 11.37 (1.0)	<0.001
SMD ≤−0.20 (at least a small effect)	18	−0.480 (−0.67 to 0.29)	z = −5.07 (23.6)	<0.001
SMD >−0.20	12	−0.097 (−0.22 to 0.03)	z = −1.77 (11.0)	0.106
**Intervention type**			χ^2^ = 13.13 (2.0)	0.001
Psychological therapy	23	−0.455 (−0.61 to −0.30)	z = −5.87 (9.8)	<0.001
Antidepressant	3	−0.236 (−0.56 to 0.09)	z = −1.42 (3.9)	0.154
Exercise	7	−0.047 (−0.25 to 0.15)	z = −0.58 (6.0)	0.583
**Mood as a primary outcome**			χ^2^ = 3.54 (1.0)	0.060
Primary	10	−0.565 (−0.86 to −0.27)	z = −3.70 (17.0)	<0.001
Secondary	23	−0.257 (−0.38 to −0.14)	z = −4.45 (22.0)	0.002
**IBD type**^b^			χ^2^ = 1.50 (1.0)	0.263
Ulcerative colitis	9	−0.160 (−0.33 to 0.01)	z = −2.12 (8.0)	0.061
Crohn’s disease	11	−0.307 (−0.51 to −0.10)	z = −2.94 (17.2)	0.003

SMD: standardised mean difference.

a Not every study had usable mood data.

b Not every study analysed Crohn’s Disease and Ulcerative Colitis separately.

The interventions included in the review significantly improved mood outcomes (SMD = −0.501, 95% CI: −0.73 to −0.27, z = −4.47, p < 0.001). There was low heterogeneity (τ^2^ < 0.001) and no evidence of publication bias (Egger’s test = 1.05, p = 0.391). 5 mood effects from 4 studies^29^^,^^31^^,^^52^^,^^54^ exceeded the DFBETA threshold; however, when these were excluded in a sensitivity analysis, there was still a significant mood effect post-intervention (SMD = −0.427, 95% CI: −0.59 to −0.27, z = −5.31, p < 0.001), with low heterogeneity (τ^2^ < 0.001) and no significant publication bias (Egger’s test = 0.692, p = 0.467). Studies were categorised based on whether they demonstrated at least a small effect on mood (see Methods). Effect on mood impacted the effect of interventions on inflammation (χ^2^ (1) = 11.37, p < 0.001); interventions with effects ≥0.2 (lower limit for small effects) showed significantly greater post-intervention effects on inflammation (SMD = −0.376, 95% CI: −0.61 to −0.15, p = 0.003). Interventions that had at least a small effect on mood had a medium-sized effect on inflammation (SMD = 0.480, 95% CI: −0.67 to −0.29, p < 0.001).

Intervention type significantly moderated the overall association (χ^2^ (2) = 13.13, p = 0.001). Psychological therapies had a significant small-medium effect on inflammatory biomarkers (SMD = −0.455, 95% CI: −0.61 to −0.30, p < 0.001), whilst antidepressant (SMD = −0.236, 95% CI: −0.56 to −0.09, p = 0.154) and exercise interventions (SMD = −0.047, 95% CI: −0.25 to 0.15, p = 0.583) were insignificant. When a mood outcome was the primary outcome for the study, there was a medium effect size on inflammatory markers (SMD = −0.565, 95% CI: −0.86 to −0.27, p = 0.001), and a small effect observed in studies where it was a secondary outcome (SMD = −0.257, 95% CI: −0.38 to −0.14, p = 0.002). However, whether mood was primary/secondary was not a significant moderator (SMD = −0.307, 95% CI: −0.65 to 0.04, χ^2^ (1) = 3.54, p = 0.060), nor was IBD subtype (χ^2^ (1) = 1.50, p = 0.263).

### Risk of bias

Risk of Bias was low in 4 (14%) studies,^41^^,^^45^^,^^49^^,^^50^ high in 18 (64%) studies^29^, ^30^, ^31^^,^^33^, ^34^, ^35^, ^36^^,^^38^^,^^39^^,^^44^^,^^46^, ^47^, ^48^^,^^51^, ^52^, ^53^, ^54^, ^55^ and showed some concerns in 6 (21%) studies.^28^^,^^32^^,^^37^^,^^40^^,^^42^^,^^43^ For one high risk study, only an abstract was available,^35^ so downgrading this study based on limited information may be unreasonable. Bias arose mostly from inadequate reporting of results: 5 (19%) studies failed to report on all inflammatory outcomes described in protocols or trial registrations^29^^,^^39^^,^^44^^,^^46^^,^^48^ and 9 (33%) were not prospectively registered.^31^^,^^33^, ^34^, ^35^^,^^47^^,^^51^, ^52^, ^53^, ^54^ See Supplementary Table S6 for Risk of Bias ratings.

## Discussion

There is accumulating evidence of the link between mental health and disease outcomes in IBD. To our knowledge, no review has investigated whether mood interventions can improve inflammatory disease activity in IBD. Across 28 RCTs (1789 participants), interventions targeting mood as an outcome significantly reduced levels of inflammation in adults with IBD when compared with controls. Individual meta-analyses were run for CRP and faecal calprotectin, both key indicators of IBD disease activity; these both demonstrated small yet statistically significant effects for mood treatments reducing inflammation. An outlier was identified in the CRP analysis, and its removal resulted in a higher effect size; however, both CRP analyses demonstrated significant, albeit small, heterogeneity, so must be interpreted with caution. No outliers were identified in the faecal calprotectin analysis, and there was no evidence of significant heterogeneity. The composite analysis, combining all inflammatory outcomes (including endoscopy, histology, white blood cells, inflammatory cytokines), showed a small significant effect of interventions for improving inflammation. The composite analysis remained significant in the sensitivity analyses when outliers were removed and when antidepressant trials were excluded.

Whilst the interventions overall showed a moderate significant effect on mood, 12 interventions failed to show even small effects, with 18 demonstrating small, medium or large effects. When these were grouped into a binary mood effect variable, effect on mood significantly moderated the relationship between intervention and inflammation. Treatments with effects of ≥0.20 for mood showed a significant, small-medium effect in improving inflammatory biomarkers, whereas ineffective treatments for mood demonstrated insignificant inflammatory effects. Psychological therapy had greater effect on biomarkers than antidepressant or exercise interventions, yielding a small-medium effect size. Although interventions that had mood outcomes as the primary outcome of the study demonstrated medium effects for inflammatory biomarkers, whether mood was primary or secondary outcome did not emerge as a significant moderator. Intervention effects did not significantly differ based on IBD subtype.

Our findings corroborate meta-analyses across long-term conditions showing enhanced immune function following psychosocial interventions,^10^^,^^11^ but add that reduced inflammation can occur specifically in IBD populations. Additional inflammatory benefit was seen in psychological therapy interventions and interventions with larger effects on mood. This suggests that the mechanism underlying the effect of psychosocial interventions on biomarkers in IBD is improved mood. Improvements in mood may directly influence immune system processes and inflammation.^56^, ^57^, ^58^ The effect may also be explained indirectly, whereby improved mental health promotes IBD self-management and self-efficacy.^59^ Future research should clarify which psychosocial constructs are most influential as well as potential indirect mechanisms and their relative contribution to reducing inflammation.

Although a previous meta-analysis found no effect of psychological interventions on self-reported clinical indices of disease activity,^16^ the current meta-analysis focussed on objective inflammatory biomarkers. This enables investigation of the possible psychoneuroimmunological mechanisms through which interventions to improve mood may alter IBD activity. For instance, the Riggott et al., 2023 meta-analysis demonstrated small effects across mood variables, and did not assess whether size of mood effect moderated the relationship with disease activity.^16^ The temporal sequence connecting psychosocial interventions to inflammation and subsequent disease activity is uncertain. The previous study finding no effect on disease activity might be due to outcome measurement being too close to therapy initiation. Timing of inflammation and disease activity may be salient and warrants further investigation.

The current meta-analysis showed small-medium improvements in mood; Riggott et al., demonstrated small effects across mood variables.^16^ Both meta-analyses found larger effects than previous review conducted in 2017,^60^ with effects negligible for depression and nonsignificant for anxiety. Progress may have been made in developing effective psychological treatments for IBD, as a recent trial has shown large effect sizes for depression/anxiety in IBD,^61^ when protocols were tailored to the needs of chronic illness patients. Moreover, in this meta-analysis, where 68% of studies were conducted since 2017, the composite effect size for mood was −0.501.

Our findings have important clinical implications: interventions for mood, particularly psychological interventions, present a strategy to improve mental health and reduce inflammation in IBD. This supports the integration of mental and physical healthcare in IBD care for improving both patient wellbeing and objective disease outcomes. This is significant as although pharmacological treatments for IBD have improved greatly in recent years, they still present some issues. For example, although infliximab is commonly used in IBD, a significant proportion of patients experience reduced treatment response following extended treatment.^62^ Many IBD medications have significant adverse side effects^63^ that are difficult for patients to manage. Indeed, medications taken to reduce inflammation are often very costly, with infliximab costing £12,584 per year^64^; whereas, an 8-week course of face-to-face CBT costs approximately £480–£800 in the UK.^65^ Given this, including psychological interventions within IBD management might confer additional benefit for inflammation levels and possibly allow for reductions in the amount of anti-inflammatory medication required. For instance, online therapist-supported CBT protocols, tailored to treat mood in long-term conditions, have been developed and tested in IBD populations, presenting an effective, acceptable, and cost-effective treatment to improve mood.^61^

### Strengths and limitations

This meta-analysis has several strengths. 1) We pre-registered the study and employed a robust and systematic approach following Cochrane guidelines^19^^,^^22^ to identify studies, extract data, and conduct analyses. 2) We used objective measures of inflammation minimising the potential for bias associated with self-reported data. 3) The robust variance estimation technique allowed inclusion of multiple immune outcomes and moderator analyses. 4) Considerable efforts were made to contact study authors to obtain continuous data (means and SDs), where only proportions or medians/ranges were reported. 5) By employing Intent-to-Treat (ITT) analyses, this study mitigates the risk of overestimating intervention effects. 6) Our large sample size of 1789 individuals, increased statistical power and precision of effect size estimates. 7) The use of Egger’s and PET-PEESE tests, to assess publication bias, revealed no significant publication bias across all effects, except the CRP analysis. 8) Sensitivity analyses (exclusion of high DFBETA values and leave-one-out meta-analysis techniques) demonstrated that effects persisted when influential points and outliers were excluded.

This study has several limitations. Meta-regression relies on aggregate level data, thus obscuring potential mediating or moderating effects at individual level, for example, age, sex, disease type or mood outcomes and preventing additional testing of mechanisms. Future research should explore psychoneuroimmunological and/or behavioural pathways that may explain this effect. Between-trial confounding was offset by our use of a robust variance meta-regression; however, unmeasured confounding within-study or within-individual may have occurred.^66^ Most included studies exhibited a high risk of bias, particularly concerning inadequate reporting of results. Variations in study follow-up assessment durations could introduce heterogeneity in the data. The scarcity of available studies reduced our power in our moderator analysis meaning that we 1) could only use three intervention categories, 2) were unable to separate specific biomarkers by disease subtype and 3) were likely underpowered to distinguish whether mood as a primary vs secondary outcome was a significant moderator.

### Conclusions

This meta-analysis adds to the growing literature on psychoneuroimmunological mechanisms in IBD prognosis and provides compelling evidence for the significant impact of interventions treating mood at reducing inflammation and improving mood in adults with IBD. This is the first review to investigate the relationship between interventions aiming to treat mood and levels of inflammatory biomarkers, including faecal calprotectin and C-Reactive Protein in IBD. The findings highlight the potential of mental health treatments as a complementary treatment for IBD patients, and future research should aim to clarify the mechanisms underlying these effects. As there is variability across intervention type, more work is needed to clarify the most effective treatment for mood in IBD as larger reductions in distress were associated with greater reductions in inflammation.

## Contributors

NS did conceptualisation, literature search, data extraction, data analysis and writing (drafting, review, and editing). JH did conceptualisation, data analysis, writing (review and editing) and risk of bias assessment. SH did literature search and risk of bias assessment. SN did data analysis, writing (review and editing). VM did conceptualisation, writing (review and editing). AJ did data extraction. RMM did conceptualisation, writing (review and editing).

## Data sharing statement

The data used in the study are available in an online repository: https://data.mendeley.com/datasets/svtmy3274p/1.

## Declaration of interests

None.
