# Supplementary material for: Do interventions for mood improve inflammatory biomarkers in inflammatory bowel disease?: a systematic review and meta-analysis
Source: eBioMedicine. 2024 Jan 24;100:104910. doi: 10.1016/j.ebiom.2023.104910 (PMC10878994; doi:10.1016/j.ebiom.2023.104910)
Supplement: Supplementary Tables S1–S6 and Figures S1–S6 [file mmc1.docx]

Supplementary Appendix

# Table of Contents

[Table of Contents 1](#_Toc149635945)

[Supplementary Table 1. The Preferred Reporting Items for Systematic Reviews and Meta-analyses (PRISMA) 2020 2](#_Toc149635946)

[Supplementary Table 2. Search strategy for meta-analysis of psychosocial/behavioural interventions effect on inflammatory biomarkers 5](#_Toc149635947)

[Supplementary Table 3. Studies excluded following full-text screening 9](#_Toc149635948)

[Supplementary Table 4. Characteristics of studies included in the meta-analysis. 10](#_Toc149635949)

[Supplementary Table 5. TIDieR table of psychosocial/behavioural intervention content. 15](#_Toc149635950)

[Supplementary Figure 1. Scatter plot of Study Effect Size and Study Average Age 19](#_Toc149635951)

[Supplementary Figure 2. Scatter plot of Study Effect Size and Study Gender Proportion 20](#_Toc149635952)

[Supplementary Figure 3. Funnel plot to assess publication bias for studies investigating the effect psychosocial/behavioural interventions on faecal calprotectin. A scatterplot of treatment effect against a measure of study precision, to visually inspect the studies for publication bias and systematic heterogeneity. 21](#_Toc149635953)

[Supplementary Figure 4. Forest plot of leave-one-out sensitivity analysis for faecal calprotectin 22](#_Toc149635954)

[Supplementary Figure 5. Funnel plot to assess publication bias for studies investigating the effect psychosocial/behavioural interventions on C-Reactive Protein. A scatterplot of treatment effect against a measure of study precision, to visually inspect the studies for publication bias and systematic heterogeneity. 23](#_Toc149635955)

[Supplementary Figure 6. Forest plot of leave-one-out sensitivity analysis for C-Reactive Protein. 24](#_Toc149635956)

[Supplementary Table 7. Risk of Bias of Included Studies 25](#_Toc149635957)

# Supplementary Table 1. The Preferred Reporting Items for Systematic Reviews and Meta-analyses (PRISMA) 2020

| **Section and Topic** | **Item #** | **Checklist item** | **Notes** |
| --- | --- | --- | --- |
| **TITLE** |  |  |  |
| Title | 1 | Identify the report as a systematic review. | Identified as a ‘Systematic Review and Meta-Analysis’ |
| **ABSTRACT** |  |  |  |
| Abstract | 2 | See the PRISMA 2020 for Abstracts checklist. | All items included. |
| **INTRODUCTION** |  |  |  |
| Rationale | 3 | Describe the rationale for the review in the context of existing knowledge. | See *Introduction*. |
| Objectives | 4 | Provide an explicit statement of the objective(s) or question(s) the review addresses. | See *Introduction*, final paragraph. |
| **METHODS** |  |  |  |
| Eligibility criteria | 5 | Specify the inclusion and exclusion criteria for the review and how studies were grouped for the syntheses. | See *Table 1. PICOS inclusion/exclusion criteria* in *Methods.* |
| Information sources | 6 | Specify all databases, registers, websites, organisations, reference lists and other sources searched or consulted to identify studies. Specify the date when each source was last searched or consulted. | See *Methods*. |
| Search strategy | 7 | Present the full search strategies for all databases, registers and websites, including any filters and limits used. | See *Eligibility criteria and selection process*. |
| Selection process | 8 | Specify the methods used to decide whether a study met the inclusion criteria of the review, including how many reviewers screened each record and each report retrieved, whether they worked independently, and if applicable, details of automation tools used in the process. | See *Eligibility criteria and selection process*. |
| Data collection process | 9 | Specify the methods used to collect data from reports, including how many reviewers collected data from each report, whether they worked independently, any processes for obtaining or confirming data from study investigators, and if applicable, details of automation tools used in the process. | See *Data extraction*. |
| Data items | 10a | List and define all outcomes for which data were sought. Specify whether all results that were compatible with each outcome domain in each study were sought (e.g. for all measures, time points, analyses), and if not, the methods used to decide which results to collect. | See *Data extraction*. |
|  | 10b | List and define all other variables for which data were sought (e.g. participant and intervention characteristics, funding sources). Describe any assumptions made about any missing or unclear information. | See *Data extraction*. |
| Study risk of bias assessment | 11 | Specify the methods used to assess risk of bias in the included studies, including details of the tool(s) used, how many reviewers assessed each study and whether they worked independently, and if applicable, details of automation tools used in the process. | See *Risk of Bias (RoB) Assessment*. |
| Effect measures | 12 | Specify for each outcome the effect measure(s) (e.g. risk ratio, mean difference) used in the synthesis or presentation of results. | See *Statistical Methods*. |
| Synthesis methods | 13a | Describe the processes used to decide which studies were eligible for each synthesis (e.g. tabulating the study intervention characteristics and comparing against the planned groups for each synthesis (item #5)). | See *Statistical Methods* and *Moderator Coding*. |
|  | 13b | Describe any methods required to prepare the data for presentation or synthesis, such as handling of missing summary statistics, or data conversions | See *Statistical Methods*. |
|  | 13c | Describe any methods used to tabulate or visually display results of individual studies and syntheses. | See *Statistical Methods*. |
|  | 13d | Describe any methods used to synthesize results and provide a rationale for the choice(s). If meta-analysis was performed, describe the model(s), method(s) to identify the presence and extent of statistical heterogeneity, and software package(s) used. | See *Statistical Methods* and *Moderator Coding*. |
|  | 13e | Describe any methods used to explore possible causes of heterogeneity among study results (e.g. subgroup analysis, meta-regression). | See *Statistical Methods* and *Moderator Coding*. |
|  | 13f | Describe any sensitivity analyses conducted to assess robustness of the synthesized results. | See *Risk of Bias (RoB) Assessment* and *Statistical Methods*. |
| Reporting bias assessment | 14 | Describe any methods used to assess risk of bias due to missing results in a synthesis (arising from reporting biases). | See *Risk of Bias (RoB) Assessment* and *Statistical Methods*. |
| Certainty assessment | 15 | Describe any methods used to assess certainty (or confidence) in the body of evidence for an outcome. | See *Risk of Bias (RoB) Assessment* and *Statistical Methods*. |
| **RESULTS** |  |  |  |
| Study selection | 16a | Describe the results of the search and selection process, from the number of records identified in the search to the number of studies included in the review, ideally using a flow diagram. | See *Study selection characteristics*. |
|  | 16b | Cite studies that might appear to meet the inclusion criteria, but which were excluded, and explain why they were excluded. | See *Study selection characteristics* and *Figure 1.* |
| Study characteristics | 17 | Cite each included study and present its characteristics. | See *Study selection characteristics* and *Supplementary material.* |
| Risk of bias in studies | 18 | Present assessments of risk of bias for each included study. | See *Risk of Bias* and *Supplementary material.* |
| Results of individual studies | 19 | For all outcomes, present, for each study: (a) summary statistics for each group (where appropriate) and (b) an effect estimate and its precision (e.g. confidence/credible interval), ideally using structured tables or plots. | See *Overall Effect Size* and *Figure 2 & 3*. |
| Results of syntheses | 20a | For each synthesis, briefly summarise the characteristics and risk of bias among contributing studies. | See *Risk of Bias* and *Supplementary material.* |
|  | 20b | Present results of all statistical syntheses conducted. If meta-analysis was done, present for each the summary estimate and its precision (e.g. confidence/credible interval) and measures of statistical heterogeneity. If comparing groups, describe the direction of the effect. | See *Overall Effect Size*. |
|  | 20c | Present results of all investigations of possible causes of heterogeneity among study results. | See *Overall Effect Size* and *Supplementary material*. |
|  | 20d | Present results of all sensitivity analyses conducted to assess the robustness of the synthesized results. | See *Overall Effect Size* and *Moderator Analyses*. |
| Reporting biases | 21 | Present assessments of risk of bias due to missing results (arising from reporting biases) for each synthesis assessed. | See *Risk of Bias* and *Supplementary material.* |
| Certainty of evidence | 22 | Present assessments of certainty (or confidence) in the body of evidence for each outcome assessed. | See *Overall Effect Size*. |
| **DISCUSSION** |  |  |  |
| Discussion | 23a | Provide a general interpretation of the results in the context of other evidence. | See *Discussion*. |
|  | 23b | Discuss any limitations of the evidence included in the review. | See *Strengths and Limitations*. |
|  | 23c | Discuss any limitations of the review processes used. | See *Discussion*. |
|  | 23d | Discuss implications of the results for practice, policy, and future research. | See *Discussion* and *Conclusions*. |
| **OTHER INFORMATION** |  |  |  |
| Registration and protocol | 24a | Provide registration information for the review, including register name and registration number, or state that the review was not registered. | See *Abstract* and *Methods*. |
|  | 24b | Indicate where the review protocol can be accessed, or state that a protocol was not prepared. | See *Methods*. |
|  | 24c | Describe and explain any amendments to information provided at registration or in the protocol. | See *Methods*. |
| Support | 25 | Describe sources of financial or non-financial support for the review, and the role of the funders or sponsors in the review. | See *Acknowledgments*. |
| Competing interests | 26 | Declare any competing interests of review authors. | See *Acknowledgments*. |
| Availability of data, code and other materials | 27 | Report which of the following are publicly available and where they can be found: template data collection forms; data extracted from included studies; data used for all analyses; analytic code; any other materials used in the review. | See *Methods* and *Results*. |

# Supplementary Table 2. Search strategy for meta-analysis of psychosocial/behavioural interventions effect on inflammatory biomarkers

| Population | Intervention | Outcome |
| --- | --- | --- |
| **EMBASE (Ovid)**   - exp Inflammatory bowel disease/ - exp Ulcerative colitis/ - exp Crohn disease/ - IBD.mp - inflammatory bowel disease*.mp - ulcerative colitis.mp - crohn* disease.mp - enteritis.mp - ileitis.mp - proctitis.mp - pancolitis.mp - enterocolitis.mp - proctocolitis.mp - colitis.mp | **EMBASE (Ovid)**   - exp Depression/ - exp Anxiety/ - exp Anxiety disorder/ - depressi*.mp - anxious*.mp - anxiety disorder*.mp - panic disorder*.mp - psychologic* stress*.mp - mood.mp - mental health.mp - psychosocial*.mp - gut AND brain.mp | **EMBASE (Ovid)**   - exp Disease activity/ - exp Disease course/ - exp Disease severity/ - exp C reactive protein/ - exp Feces analysis/ - exp Cytokine/ - exp hypothalamus hypophysis adrenal system/ - exp Hemoglobin/ - exp gastrointestinal endoscopy/ - exp tumor necrosis factor/ - exp interferon/ - exp chemokine/ - exp toll like receptor/ - exp neutrophil/ - exp macrophage/ - exp monocyte/ - exp lymphocyte/ - exp natural killer cell/ - exp hydrocortisone/ - CRP.mp - C reactive protein.mp - f?ecal calprotectin.mp - cytokine*.mp - pro-inflammat*.mp - interleukin.mp - inflammat*.mp - endoscop*.mp - disease activity.mp - clinical activity.mp - h?ematocrit.mp - h?emoglobin.mp - albumin.mp - HPA axis.mp - hypothalamus pituitary adrenal axis.mp - interferon.mp - “tumor necrosis factor”.mp - Chemokine.mp - “toll-like receptor”.mp - NF-kB.mp - Neutrophil.mp - Macrophage.mp - Monocyte.mp - Lymphocyte.mp - “natural killer cell”.mp - “NK cell”.mp - “polymorphonuclear leukocytes”.mp - Cortisol.mp |
| **MEDLINE (Ovid)**   - exp Inflammatory bowel diseases/ - exp Colitis, ulcerative/ - exp Crohn disease/ - IBD.mp - inflammatory bowel disease*.mp - ulcerative colitis.mp - crohn* disease.mp - enteritis.mp - ileitis.mp - proctitis.mp - pancolitis.mp - enterocolitis.mp - proctocolitis.mp - colitis.mp | **MEDLINE (Ovid)**   - exp Depression/ - exp Depressive disorder/ - exp Anxiety/ - exp Anxiety disorders/ - exp Stress, psychological/ - depressi*.mp - anxious*.mp - anxiety disorder*.mp - panic disorder*.mp - psychologic* stress*.mp - mood.mp - mental health.mp - psychosocial*.mp - gut AND brain.mp | **MEDLINE (Ovid)**   - exp C-reactive protein/ - exp Cytokines/ - exp Pituitary-adrenal system/ - exp Hypothalamo-Hypophyseal System/ - exp Hemoglobins/ - exp endoscopy, gastrointestinal/ - exp interferons/ - exp Tumor Necrosis Factor-alpha/ - exp chemokines/ - exp Toll-Like Receptors/ - exp NF-kappa B/ - exp Neutrophils/ - exp Macrophages/ - exp Monocytes/ - exp Lymphocytes/ - exp Killer Cells, Natural/ - exp Hydrocortisone/ - CRP.mp - C reactive protein.mp - f?ecal calprotectin.mp - cytokine*.mp - pro-inflammat*.mp - interleukin.mp - inflammat*.mp - endoscop*.mp - disease activity.mp - clinical activity.mp - h?ematocrit.mp - h?emoglobin.mp - albumin.mp - HPA axis.mp - hypothalamus pituitary adrenal axis.mp - interferon.mp - “tumor necrosis factor”.mp - Chemokine.mp - “toll-like receptor”.mp - NF-kB.mp - Neutrophil.mp - Macrophage.mp - Monocyte.mp - Lymphocyte.mp - “natural killer cell”.mp - “NK cell”.mp - “polymorphonuclear leukocytes”.mp - Cortisol.mp |
| **Global Health (Ovid)**   - exp Inflammatory bowel diseases/ - exp Crohn's disease/ - exp Ulcerative colitis/ - IBD.mp - inflammatory bowel disease*.mp - ulcerative colitis.mp - crohn* disease.mp - enteritis.mp - ileitis.mp - proctitis.mp - pancolitis.mp - enterocolitis.mp - proctocolitis.mp - colitis.mp | **Global Health (Ovid)**   - exp depression/ - exp anxiety/ - depressi*.mp - anxious*.mp - anxiety disorder*.mp - panic disorder*.mp - psychologic* stress*.mp - mood.mp - mental health.mp - psychosocial*.mp - gut AND brain.mp | **Global Health (Ovid)**   - exp Disease markers/ - exp Disease course/ - exp Cytokines/ - exp Haemoglobin/ - exp interferons/ - exp tumour necrosis factor/ - exp Chemokines/ - exp toll-like receptors/ - exp NF-kappa B/ - exp Neutrophils/ - exp macrophages/ - exp Monocytes/ - exp lymphocytes/ - exp natural killer cells/ - exp leukocytes/ - exp hydrocortisone/ - CRP.mp - C reactive protein.mp - f?ecal calprotectin.mp - cytokine*.mp - pro-inflammat*.mp - interleukin.mp - inflammat*.mp - endoscop*.mp - disease activity.mp - clinical activity.mp - h?ematocrit.mp - h?emoglobin.mp - albumin.mp - HPA axis.mp - hypothalamus pituitary adrenal axis.mp - interferon.mp - “tumor necrosis factor”.mp - Chemokine.mp - “toll-like receptor”.mp - NF-kB.mp - Neutrophil.mp - Macrophage.mp - Monocyte.mp - Lymphocyte.mp - “natural killer cell”.mp - “NK cell”.mp - “polymorphonuclear leukocytes”.mp - Cortisol.mp |
| **APA Psych (Ovid)**   - IBD.mp - inflammatory bowel disease*.mp - ulcerative colitis.mp - crohn* disease.mp - enteritis.mp - ileitis.mp - proctitis.mp - pancolitis.mp - enterocolitis.mp - proctocolitis.mp - colitis.mp | **APA Psych (Ovid)**   - exp Major depression/ - exp Depression (emotion)/ - exp Anxiety disorders/ - exp Anxiety/ - exp Mental disorders due to general medical conditions/ - exp Comorbidity/ - exp Psychiatric symptoms/ - exp Psychological stress/ - exp Perceived stress/ - exp psychosocial factors/ - depressi*.mp - anxious*.mp - anxiety disorder*.mp - panic disorder*.mp - psychologic* stress*.mp - mood.mp - mental health.mp - psychosocial*.mp - gut AND brain.mp | **APA Psych (Ovid)**   - exp Disease course/ - exp Disease progression/ - exp Relapse (Disorders)/ - exp Remission (Disorders)/ - exp Cytokines/ - exp Hypothalamic Pituitary Adrenal Axis/ - exp interferons/ - exp leucocytes/ - exp interleukins/ - exp tumor necrosis factor/ - exp lymphocytes/ - exp natural killer cells/ - exp hydrocortisone/ - CRP.mp - C reactive protein.mp - f?ecal calprotectin.mp - cytokine*.mp - pro-inflammat*.mp - interleukin.mp - inflammat*.mp - endoscop*.mp - disease activity.mp - clinical activity.mp - h?ematocrit.mp - h?emoglobin.mp - albumin.mp - HPA axis.mp - hypothalamus pituitary adrenal axis.mp - interferon.mp - “tumor necrosis factor”.mp - Chemokine.mp - “toll-like receptor”.mp - NF-kB.mp - Neutrophil.mp - Macrophage.mp - Monocyte.mp - Lymphocyte.mp - “natural killer cell”.mp - “NK cell”.mp - “polymorphonuclear leukocytes”.mp - Cortisol.mp |
| **Web of Science**  ALL=IBD  ALL=inflammatory bowel disease*  ALL=Crohn* disease  ALL=ulcerative colitis  ALL=enteritis  ALL=ileitis  ALL=proctitis  ALL=pancolitis  ALL=enterocolitis  ALL=proctocolitis  ALL=colitis | **Web of Science**  ALL=depress*  ALL=anxiety  ALL=anxious*  ALL=panic disorder*  ALL=psychologic* stress*  ALL=psychosocial*  ALL=mood  ALL=mental health  ALL=(gut AND brain) | **Web of Science**  ALL=CRP  ALL=C*reactive protein  ALL=f*ecal calprotectin  ALL=cytokine*  ALL=pro-inflammat*  ALL=interleukin  ALL=inflammat*  ALL=endoscop*  ALL=disease activity  ALL=clinical activity  ALL=h*ematocrit  ALL=h*emoglobin  ALL=albumin  ALL=HPA axis  ALL=hypothalamus pituitary adrenal axis  ALL=interferon  ALL=tumo*r necrosis factor  ALL=chemokine*  ALL=toll-like receptor  ALL=NF-kappa B  ALL=NF-kB  ALL=Neutrophil  ALL=macrophage  ALL=monocyte  ALL=lymphocyte  ALL=leu*ocyte  ALL=natural killer cell  ALL=NK cell  ALL=polymorphonuclear leu*ocytes  ALL=cortisol |

# Supplementary Material 1. Syntax for STATA v17.0

** install relevant packages

ssc install metaeff

ssc install metaan

ssc install admetan

ssc install metaeff

ssc install metareg

ssc install metafunnel

ssc install robumeta

ssc install ipdmetan

ssc install metabias

ssc install center

ssc install coefplot

* compute the DFBETA values to investigate influence in model

metan effectsize_direction SEeffectsize, random label(namevar=es_id) boxsca(75) texts(100)xlabel(-1 -.5 .5 1) favours (Intervention # Control)

metareg _ES, wsse(_seES) reml

local b = _b[_cons]

gen b_loo = .

gen db_loo = .

gen se_loo = .

local N = _N

quietly {

      forv n = 1/`N' {

            metareg effectsize_direction if _n!=`n', wsse(_seES) reml

            replace b_loo = _b[_cons] in `n'

            replace db_loo = `b'-_b[_cons] in `n'

            replace se_loo = _se[_cons] in `n'

      }

}

gen dfbeta = db_loo/se_loo

gen hidfb = abs(dfbeta)>(2/sqrt(_N)) // indicator for high dfbetas

gsort -dfbeta

list Reference effectsize_direction _seES b_loo db_loo se_loo dfbeta hidfb, noobs sep(0)

**** Composite biomarker analysis – robust variance meta-regression ****

*assess whether changing assumed correlation rho has impact on size or direction of effect

foreach val of numlist 0(.1).9 {

robumeta effectsize, study(studyid) variance(SEeffectsize) weighttype(random) rho(`val')

}

*main effect for composite biomarker analysis. Cluster Effect Size by study with recommended rho

robumeta effectsize_direction if duplicate_data==0, study(studyid) variance(var) weighttype(random) rho(.80)

lincom _b[_cons]

*values for overall main effect forest plot

xi: metareg effectsize_direction i.studyid if duplicate_data==0, wsse(SEeffectsize)

margins , by(studyid) post

coefplot

*assessing publication bias in the main effect

// FAT-PET-PEESE

            gen prec  = 1/SEeffectsize

            gen prec2 = (prec)^2

            gen tvalue = effectsize_direction/SEeffectsize

noisily {

      * random effect estimate

      metareg effectsize_direction if duplicate_data==0, wsse(SEeffectsize) mm

      local bRE = _b[_cons]

      local sRE = _se[_cons]

      test _b[_cons] = 0

      local pRE = r(p)

      * PET estimate of publication bias (same estimate as Egger test)

      gen constant = 1

      reg tvalue prec constant if duplicate_data==0, noc vce(cluster studyid)

      local bFAT = _b[constant]

      local sFAT = _se[constant]

      test _b[constant] = 0

      local pFAT = r(p)

      local bPET = _b[prec]

      local sPET = _se[prec]

      test prec = 0

      local pPET = r(p)

      drop constant

      * PEESE estimate of publication bias

      reg tvalue prec SEeffectsize if duplicate_data==0, noc vce(cluster studyid)

      local bPEESE = _b[prec]

      local sPEESE = _se[prec]

      test prec = 0

      local pPEESE = r(p)

}

di "effect unadjusted (RE) = " %5.3f `bRE' " ( SE = " %5.3f `sRE' ") ; p = " %5.3f `pRE'

di "Egger bias [FAT] = " %5.3f `bFAT' " ( SE = " %5.3f `sFAT' ") ; p = " %5.3f `pFAT'

di "effect adjusted [PEESE] = " %5.3f `bPEESE' " ( SE = " %5.3f `sPEESE' ") ; p = " %5.3f `pPEESE'

di "effect adjusted [PET] = " %5.3f `bPET' " ( SE = " %5.3f `sPET' ") ; p = " %5.3f `pPET'

*** Sensitivity analyses

** sensitivity analysis without outliers

*main effect

robumeta effectsize_direction if duplicate_data==0 & hidfb==0, study(studyid) variance(var) weighttype(random) rho(.80)

lincom _b[_cons]

list Reference es_id if hidfb==1

*FAT-PET-PEESE and Eggers without high DFBETA

noisily {

      * random effect estimate

      metareg effectsize_direction if duplicate_data==0 & hidfb==0, wsse(SEeffectsize) mm

      local bRE = _b[_cons]

      local sRE = _se[_cons]

      test _b[_cons] = 0

      local pRE = r(p)

      * PET estimate of publication bias (same estimate as Egger test)

      gen constant = 1

      reg tvalue prec constant if duplicate_data==0 & hidfb==0, noc vce(cluster studyid)

      local bFAT = _b[constant]

      local sFAT = _se[constant]

      test _b[constant] = 0

      local pFAT = r(p)

      local bPET = _b[prec]

      local sPET = _se[prec]

      test prec = 0

      local pPET = r(p)

      drop constant

      * PEESE estimate of publication bias

      reg tvalue prec SEeffectsize if duplicate_data==0 & hidfb==0, noc vce(cluster studyid)

      local bPEESE = _b[prec]

      local sPEESE = _se[prec]

      test prec = 0

      local pPEESE = r(p)

}

di "effect unadjusted (RE) = " %5.3f `bRE' " ( SE = " %5.3f `sRE' ") ; p = " %5.3f `pRE'

di "Egger bias [FAT] = " %5.3f `bFAT' " ( SE = " %5.3f `sFAT' ") ; p = " %5.3f `pFAT'

di "effect adjusted [PEESE] = " %5.3f `bPEESE' " ( SE = " %5.3f `sPEESE' ") ; p = " %5.3f `pPEESE'

di "effect adjusted [PET] = " %5.3f `bPET' " ( SE = " %5.3f `sPET' ") ; p = " %5.3f `pPET'

** sensitivity analysis controlling for age and gender

robumeta effectsize_direction averageage if duplicate_data==0, study(studyid) variance(var) weighttype(random) rho(.80)

lincom _b[_cons]

robumeta effectsize_direction sexratio if duplicate_data==0, study(studyid) variance(var) weighttype(random) rho(.80)

lincom _b[_cons]

** sensitivity analysis without antidepressant trials

*main effect

preserve

drop if iv_type==5

robumeta effectsize_direction if duplicate_data==0, study(studyid) variance(var) weighttype(random) rho(.80)

*FAT-PET-PEESE and Eggers without antidepressant trials

noisily {

      * random effect estimate

      metareg effectsize_direction if duplicate_data==0, wsse(SEeffectsize) mm

      local bRE = _b[_cons]

      local sRE = _se[_cons]

      test _b[_cons] = 0

      local pRE = r(p)

      * PET estimate of publication bias (same estimate as Egger test)

      gen constant = 1

      reg tvalue prec constant if duplicate_data==0, noc vce(cluster studyid)

      local bFAT = _b[constant]

      local sFAT = _se[constant]

      test _b[constant] = 0

      local pFAT = r(p)

      local bPET = _b[prec]

      local sPET = _se[prec]

      test prec = 0

      local pPET = r(p)

      drop constant

      * PEESE estimate of publication bias

      reg tvalue prec SEeffectsize if duplicate_data==0, noc vce(cluster studyid)

      local bPEESE = _b[prec]

      local sPEESE = _se[prec]

      test prec = 0

      local pPEESE = r(p)

}

di "effect unadjusted (RE) = " %5.3f `bRE' " ( SE = " %5.3f `sRE' ") ; p = " %5.3f `pRE'

di "Egger bias [FAT] = " %5.3f `bFAT' " ( SE = " %5.3f `sFAT' ") ; p = " %5.3f `pFAT'

di "effect adjusted [PEESE] = " %5.3f `bPEESE' " ( SE = " %5.3f `sPEESE' ") ; p = " %5.3f `pPEESE'

di "effect adjusted [PET] = " %5.3f `bPET' " ( SE = " %5.3f `sPET' ") ; p = " %5.3f `pPET'

ipdmetan effectsize_direction SEeffectsize if duplicate_data==0 & biocat_id==6, study(Reference) xlabel(-1 -.5 .5 1) favours (Intervention # Control) sortby(effectsize_direction)

ipdmetan effectsize_direction SEeffectsize  if duplicate_data==0 & biocat_id==3, study(Reference) xlabel(-1 -.5 .5 1) favours (Intervention # Control) sortby(effectsize_direction)

restore

**** Individual biomarkers – CRP & FCP ****

***FCP

*FCP main effect and forest plot

metan effectsize_direction SEeffectsize if duplicate_data==0 & biocat_id==6, random label(namevar=Reference) boxsca(75) texts(100)xlabel(-1 -.5 .5 1) favours (Intervention # Control) sortby(effectsize_direction)

*FC egger test

//    egger test

metabias effectsize_direction SEeffectsize if duplicate_data==0 & biocat_id==6, egger graph

metafunnel effectsize_direction SEeffectsize if duplicate_data==0 & biocat_id==6, xtitle(Effect Size) ytitle(SE of Effect Size)

* FC egger test and FAT-PET-PEESE

// FAT-PET-PEESE

noisily {

      * random effect estimate

      metareg effectsize_direction if duplicate_data==0 & biocat_id==6, wsse(SEeffectsize) mm

      local bRE = _b[_cons]

      local sRE = _se[_cons]

      test _b[_cons] = 0

      local pRE = r(p)

      * PET estimate of publication bias (same estimate as Egger test)

      gen constant = 1

      reg tvalue prec constant if duplicate_data==0 & biocat_id==6, noc vce(robust)

      local bFAT = _b[constant]

      local sFAT = _se[constant]

      test _b[constant] = 0

      local pFAT = r(p)

      local bPET = _b[prec]

      local sPET = _se[prec]

      test prec = 0

      local pPET = r(p)

      drop constant

      * PEESE estimate of publication bias

      reg tvalue prec SEeffectsize if duplicate_data==0 & biocat_id==6, noc vce(robust)

      local bPEESE = _b[prec]

      local sPEESE = _se[prec]

      test prec = 0

      local pPEESE = r(p)

}

di "effect unadjusted (RE) = " %5.3f `bRE' " ( SE = " %5.3f `sRE' ") ; p = " %5.3f `pRE'

di "Egger bias [FAT] = " %5.3f `bFAT' " ( SE = " %5.3f `sFAT' ") ; p = " %5.3f `pFAT'

di "effect adjusted [PEESE] = " %5.3f `bPEESE' " ( SE = " %5.3f `sPEESE' ") ; p = " %5.3f `pPEESE'

di "effect adjusted [PET] = " %5.3f `bPET' " ( SE = " %5.3f `sPET' ") ; p = " %5.3f `pPET'

**FC sensitivity analysis

preserve

keep if biocat_id==6 & duplicate_data==0

* dfbetas and sensitivity analysis rerun as metareg

metareg _ES, wsse(_seES) reml

local b = _b[_cons]

local N = _N

gsort -dfbeta

list ref effectsize_direction _seES b_loo db_loo se_loo dfbeta hidfb, noobs sep(0)

*FCP leave-one-out meta-analysis plot

gsort -dfbeta

encode refonly, gen(i)

gen ll_loo = b_loo+invnormal(.025)*se_loo

gen ul_loo = b_loo+invnormal(.975)*se_loo

gen lab = string(dfbeta, "%5.2f")

replace lab = lab+"*" if hidfb==1

local N = _N

tw (scatter i b_loo, msymbol(O) mcolor(gs10) mlab(lab) mlabpos(1) mlabc(black) mlabsize(vsmall)) ///

   (rspike ll_loo ul_loo i, horizontal lcolor(gs10)) ///

   , title("Leave-one-out sensitivity analysis") ///

   ylab(1(1)`N', val angle(0)) ytitle("") ///

   xtitle("Pooled effect") xlab(, nogrid) xline(-.187, lcolor(gs12)) xlab(-1(.5).5) ///

   legend(off) note("Note: Dashed line is the overall pooled effect; Marker values are DFBETAs (*=influential)") ///

   name(FCloo, replace) xsize(10) ysize(8)

restore

*** CRP

*CRP main effect and forest plot

metan effectsize_direction SEeffectsize if duplicate_data==0 & biocat_id==3, random label(namevar=Reference) boxsca(75) texts(100)xlabel(-1 -.5 .5 1) favours (Intervention # Control) sortby(effectsize_direction)

* CRP egger test and FAT-PET-PEESE

//    egger test

metabias effectsize_direction SEeffectsize if duplicate_data==0 & biocat_id==3, egger graph

metafunnel effectsize_direction SEeffectsize if duplicate_data==0 & biocat_id==3, xtitle(Effect Size) ytitle(SE of Effect Size)

// FAT-PET-PEESE

noisily {

      * random effect estimate

      metareg effectsize_direction if duplicate_data==0 & biocat_id==3, wsse(SEeffectsize) mm

      local bRE = _b[_cons]

      local sRE = _se[_cons]

      test _b[_cons] = 0

      local pRE = r(p)

      * PET estimate of publication bias (same estimate as Egger test)

      gen constant = 1

      reg tvalue prec constant if duplicate_data==0 & biocat_id==3, noc vce(robust)

      local bFAT = _b[constant]

      local sFAT = _se[constant]

      test _b[constant] = 0

      local pFAT = r(p)

      local bPET = _b[prec]

      local sPET = _se[prec]

      test prec = 0

      local pPET = r(p)

      drop constant

      * PEESE estimate of publication bias

      reg tvalue prec SEeffectsize if duplicate_data==0 & biocat_id==3, noc vce(robust)

      local bPEESE = _b[prec]

      local sPEESE = _se[prec]

      test prec = 0

      local pPEESE = r(p)

}

di "effect unadjusted (RE) = " %5.3f `bRE' " ( SE = " %5.3f `sRE' ") ; p = " %5.3f `pRE'

di "Egger bias [FAT] = " %5.3f `bFAT' " ( SE = " %5.3f `sFAT' ") ; p = " %5.3f `pFAT'

di "effect adjusted [PEESE] = " %5.3f `bPEESE' " ( SE = " %5.3f `sPEESE' ") ; p = " %5.3f `pPEESE'

di "effect adjusted [PET] = " %5.3f `bPET' " ( SE = " %5.3f `sPET' ") ; p = " %5.3f `pPET'

**CRP sensitivity analysis

preserve

keep if biocat_id==3 & duplicate_data==0

* dfbetas and sensitivity analysis rerun as metareg

metareg _ES, wsse(_seES) reml

local b = _b[_cons]

local N = _N

gsort -dfbeta

list Reference effectsize_direction _seES b_loo db_loo se_loo dfbeta hidfb, noobs sep(0)

*CRP leave-one-out meta-analysis plot

gsort -dfbeta

encode refonly, gen(i)

gen ll_loo = b_loo+invnormal(.025)*se_loo

gen ul_loo = b_loo+invnormal(.975)*se_loo

gen lab = string(dfbeta, "%5.2f")

replace lab = lab+"*" if hidfb==1

local N = _N

tw (scatter i b_loo, msymbol(O) mcolor(gs10) mlab(lab) mlabpos(1) mlabc(black) mlabsize(vsmall)) ///

   (rspike ll_loo ul_loo i, horizontal lcolor(gs10)) ///

   , title("Leave-one-out sensitivity analysis") ///

   ylab(1(1)`N', val angle(0)) ytitle("") ///

   xtitle("Pooled effect") xlab(, nogrid) xline(-.413, lcolor(gs12)) xlab(-1(.5).5) ///

   legend(off) note("Note: Dashed line is the overall pooled effect; Marker values are DFBETAs (*=influential)") ///

   name(CRPloo, replace) xsize(10) ysize(8)

restore

**** Moderator analysis

** Effect of mood, with a minimum SMD of 0.2

robumeta effectsize_direction moodeff_binary if duplicate_data==0, study(studyid) variance(var) weighttype(random) rho(.80)

lincom _b[_cons]+_b[moodeff_binary]

mat li e(b)

testparm moodeff_binary

** Intervention type, antidepressant, psychotherapy and exercise

robumeta effectsize_direction iv_psychtheory iv_antidepressant if duplicate_data==0, study(studyid) variance(var) weighttype(random) rho(.80)

lincom _b[_cons]+_b[iv_psychtheory]

lincom _b[_cons]+_b[iv_antidepressant]

testparm iv_exercise iv_psychtheory iv_antidepressant

**Mood as a primary or secondary outcome

robumeta effectsize_direction mood_primary if duplicate_data==0, study(studyid) variance(var) weighttype(random) rho(.80)

lincom _b[_cons]+_b[mood_primary]

mat li e(b)

testparm mood_primary

** IBD disease subtype

*Where studies report IBD totals, as well as CD & UC, delete the IBD effect sizes

preserve

drop if studyid==1005

drop if studyid==1018

robumeta effectsize_direction type_cd if type_ibd==0, study(studyid) variance(var) weighttype(random) rho(.80)

lincom _b[_cons]+_b[type_cd]

testparm type_cd type_uc

restore

# Supplementary Table 3. Studies excluded following full-text screening

| **Studies excluded because authors did not respond**   1. Boye B, Lundin KE, Jantschek G, Leganger S, Mokleby K, Tangen T, et al. INSPIRE study: does stress management improve the course of inflammatory bowel disease and disease-specific quality of life in distressed patients with ulcerative colitis or Crohn's disease? A randomized controlled trial. Inflammatory Bowel Diseases. 2011;17(9):1863-73. 2. McNelly A, Nathan I, Monte M, Grimble G, Norton C, Bredin F, et al. Inflammatory bowel disease and fatigue: The effect of physical activity and/or omega 3 supplementation. Gut. 2015;1):A88. 3. Sajadinezhad M, Adibi P, Haghjoo S. Psychological treatments on UC patients' psychological and somatic symptoms. Journal of Gastroenterology and Hepatology. 2013;3):365. 4. Seeger WA, Thieringer J, Allmendinger B, Esters P, Schulze H, Dignass A. Moderate Endurance- and Muscle Trainining Is Safe to Perform for Patients with Quiescent or Mild Active Crohns Disease and Increases Their Strength. Gastroenterology. 2020;158(6 Supplement 1):S-426. 5. Vogelaar L, van't Spijker A, Timman R, van Tilburg AJ, Bac D, Vogelaar T, et al. Fatigue management in patients with IBD: a randomised controlled trial. Gut. 2014;63(6):911-8. |
| --- |
| **Studies excluded because they were ongoing**   1. Lowe B, Nestoriuc Y, Andresen V, Vettorazzi E, Zapf A, Hubener S, et al. Persistence of gastrointestinal symptoms in irritable bowel syndrome and ulcerative colitis: study protocol for a three-arm randomised controlled trial (SOMA.GUT-RCT). Bmj Open. 2022;12(6). 2. Norton C, Syred J, Kerry S, Artom M, Sweeney L, Hart A, Czuber-Dochan W, Taylor SJ, Mihaylova B, Roukas C, Aziz Q. Supported online self-management versus care as usual for symptoms of fatigue, pain and urgency/incontinence in adults with inflammatory bowel disease (IBD-BOOST): study protocol for a randomised controlled trial. Trials. 2021 Dec;22(1):1-8. 3. Ter Avest MM, van Velthoven AS, Speckens AE, Dijkstra G, Dresler M, Horjus CS, Römkens TE, Witteman EM, van Dop WA, Bredero QM, Nissen LH. Effectiveness of Mindfulness-Based Cognitive Therapy in reducing psychological distress and improving sleep in patients with Inflammatory Bowel Disease: study protocol for a multicentre randomised controlled trial (MindIBD). BMC psychology. 2023 Dec;11(1):1-2. |

|  |
| --- |

# Supplementary Table 4. Characteristics of studies included in the meta-analysis.

| **Reference; country** | **Intervention type** | **Control group** | **Sample size** | **IBD type** | **Intervention length** (weeks) | **Inflammatory markers** | **Follow-up time point** (months) | **Age mean (SD)** | | **Sex n (%female)** | | **Ethnicity** | | **Inclusion criteria:** mood disorder; disease activity |
| --- | --- | --- | --- | --- | --- | --- | --- | --- | --- | --- | --- | --- | --- | --- |
|  |  |  |  |  |  |  |  | **active** | **control** | **active** | **control** | **active** | **control** |  |
| Berrill et al., 2014; UK | Mindfulness & CBT | CAU | 66 | IBD | 16 weeks | FC | 4 | 44.4 (11.7) | 45.4 (10.6) | 25 (76%) | 26 (79%) | not reported | | remission ("adapted disease activity index") and CRP <10 mg/L;  presence of IBS-type symptoms or a high perceived stress level |
| Cramer et al., 2017; Germany | Yoga | CAU | 77 | UC | 12 weeks | leucocytes, ESR, CRP, FL, FC | 3 | 45 (13.3) | 46.1 (10.4) | 28 (72%) | 30 (79%) | not reported | | Clinical remission for 4 - 52 weeks (RI ≤ 4); impaired QoL (IBDQ < 170) |
| Cronin et al., 2019; Ireland | Exercise | CAU | 14 | IBD | 8 weeks | CRP, IL-10, IL-6, IL-8, TNF-α | 2 | 32.57 (3.51) | 32.71 (2.75) | 4 (31%) | 1 (14.3%) | not reported | | Disease remission (definition not reported) - |
| Elsenbruch et al., 2005; Germany | Mind Body Therapy (Stress Management, Exercise & Diet advice, CBT) | CAU | 30 | UC | 10 weeks | cortisol, leucocytes, granulocytes, lymphocytes, monocytes | 2.5 | 42.9 (8.6) | 42.4 (11.4) | 10 (67%) | 10 (67%) | not reported | | - - |
| Ewais et al., 2021 * Australia | MBCT | CAU | 58 | IBD | 8 weeks | IL-6, FC, ESR, CRP | 2 | 22.1 (3.1) | 23.2 (3.4) | 18 (56%) | 17 (65%) | not reported | | -  Included clinical diagnosis of depression and DASS-d ≥10, excluded severe depression and other mental illness. |
| Gavrilescu et al., 2020; Romania | CBT & Psychoeducation | CAU | 60 | IBD | 24 weeks | FC | 12 | 43.27 (14.21) | 44.67 (11.16) | 10 (33%) | 13 (43%) | not reported | | - - |
| Gerbarg et al., 2015; USA | Breathing, Movement and Meditation | Waitlist control including Psychoeducation (time and attention) | 29 | IBD | 2 days | CRP, FC | 6 | 49.27 (14.21) | 58.57 (16.22) | 7 (47%) | 10 (71%) | not reported | | -  - |
| Gonzalez-Moret et al., 2020; Spain | Mindfulness | CAU | 57 | IBD | 8 weeks | FC, CRP, cortisol | 6 | 46.2 (10.9) | 46.3 (11.9) | 29 (78%) | 9 (45%) | not reported | | Clinical remission for 3-12 months (UC: partial Mayo Score of ≤2 points and with no item scores >1; CD: HBI <5 points). Excluded psychiatric disorders and recent emotional shock (e.g. death of a relative or an accident) |
| Goren et al., 2020; Israel | CBT & MBSR | Waitlist control | 48 | CD | 12 weeks | cortisol, IL-10, TNF-α | 3 | 31.4 (9.2) | 31.8 (9.4) | 16 (70%) | 14 (56%) | not reported | | 5-15 on HBI - |
| Goren et al., 2022; Israel | CBT & MBSR | Waitlist control | 116 | CD | 12 weeks | CRP, FC | 3 | 33.6 (13) | 32.4 (11) | 38 (69%) | 37 (61%) | not reported | | Mild-severe disease activity (HBI 5-16) included - |
| Jedel et al., 2014; USA | MBSR | Psychoeducation (time and attention) | 55 | UC | 8 weeks | FC, IL-6, IL-8, CRP, cortisol | 2 | 46.04 (12.8) | 39.68 (11.06) | 12 (44%) | 19 (68%) | Asian: 1  Black: 0  White: 26  Hispanic: 3 | Asian: 1  Black: 5  White: 22  Hispanic: 2 | In remission (Mayo UC-DAI: <2, sigmoidoscopy score: 0/1, bleeding score: 0/1), but flare in last 6 months and documented moderate severity (Mayo UC-DAI: 6–12)  - |
| Jedel et al., 2022; USA | MBSR | Psychoeducation (time and attention) | 43 | UC | 8 weeks | endoscopy (Mayo), histology (Geboes), deep, FC, IL-6, IL-8, CRP, cortisol | 2 | 44.8 (13.5) | 38.7 (10.5) | 9 (50%) | 13 (65%) | Black: 2 White: 16 Hispanic: 1 Other: 0 | Black: 2 White: 17 Hispanic: 4 Other: 1 | inclusions: inactive (Mayo ≤ 2 including endoscopy score of 0 or 1), Excluded: severe psychiatric/personality disorders |
| Klare et al., 2015; Germany | Exercise (moderate intensity) | CAU | 30 | IBD | 10 weeks | leucocytes, CRP, FC | 2.5 | 39.7 (14.7) | 42.5 (13.9) | 12 (80%) | 10 (67%) | not reported | | Remission (CDAI<220 or RI<11) - |
| Langhorst et al., 2020; Germany | Lifestyle modification (exercise, yoga, stress management, communication, habits, home remedies, cooking classes) | Psychoeducation (time and attention) | 97 | UC | 10 weeks | endoscopy (RI), histology (Riley), FL, FC, CRP | 3 | 50.3 (11.9) | 45.54 (12.49) | 34 (72%) | 35 (70%) | not reported | | Remission (RI ≤ 4) for 12M or less Impaired QoL (IBDQ<170); Excluded severe psychological illness (depression, addiction, schizophrenia) |
| Langhorst et al., 2022; Germany | Lifestyle modification & CBT & MBSR | Waitlist control including psychoeducation | 35 | CD | 10 weeks | FL | 2.5 | 47.4 (14.1) | 49.1 (10.2) | 12 (63%) | 10 (63%) | not reported | | - Excluded severe mental illness (e.g., major depression, addiction, schizophrenia) |
| Liang et al., 2022; China | Antidepressant (Venlafaxine) | Placebo | 45 | IBD | 24 weeks | CRP, ESR, TNF-α, IL-10 | 6 | 39.76(11.30) | 40.20(13.06) | 11 (44%) | 9 (45%) | not reported | | Mild-severe activity (CDAI ≥ 150; Mayo≥3) HADS of ≥8 on either/both subscales |
| March-Lujan et al., 2021; Spain | Guided Imagery and Music Therapy | Placebo | 43 | IBD | 8 weeks | hair cortisol, urinary cortisol, IgA | 2 | median (range) 50.5 (28–69) | median (range) 46.0 (20–60) | 13 (59%) | 11 (52%) | not reported | | Remission (UC: partial Mayo index < 2 points; CD: HBI< 5) - |
| Mikocka-Walus et al., 2015; Australia | CBT | CAU | 174 | IBD | 10 weeks | CRP, WBC | 6 | 46.5 (15.7) | 51.9 (16.9) | 50 (56%) | 30 (36%) | not reported | | Remission or mild for 3 months (clinical notes) Excluded serious mental illness |
| Mikocka-Walus et al., 2017; Australia | Antidepressant (Fluoxetine) | Placebo | 26 | CD | 24 weeks | FC, T Cell populations (n=12), IFN-gamma, IL-2, IL-4, IL-5, IL-6, IL-10, IL-13, TNF-α | 6 | 38.07 (13.6) | 36.67 (13.2) | 6 (43%) | 6 (50%) | not reported | | Remission (CDAI<150) for 0-12 months.  Excluded: serious uncontrolled mental illness |
| Peerani et al., 2022; Canada | Multicomponent Stress Reduction (mindfulness, yoga, CBT, positive psychology) | Waitlist control including motivational emails and count down | 101 | IBD | 12 weeks | CRP, IL-6, IL-10, TNF-α | 3 | 45.4 (14.0) | 39.7 (13.8) | 36 (74%) | 40 (77%) | Not reported | | - Included elevated stress (PSS ⩾ 8), excluded severe depression (HADS-d >10) |
| Sharma et al., 2015; India | Yoga | CAU | 100 | IBD | 8 weeks | sIL-2R, ECP | 2 | not reported | | not reported | | not reported | | Clinical remission (UC: Truelove & Witts (1955) remission; CD: CDAI≤150) Excluded patients on psychiatric medication |
| Tew et al., 2019a; UK | Exercise (high intensity interval training) | CAU | 24 | CD | 12 weeks | FC | 3 | 37.0 (11.1) | 35.0 (10.0) | 6 (46%) | 4 (36%) | White: 10 Other: 3 | White: 7 Other: 4 | Remission or mild activity (FC < 250 μg/g and CDAI <220) - |
| Tew et al., 2019b; UK | Exercise (moderate intensity) | CAU | 23 | CD | 12 weeks | FC | 3 | 38.5 (13.0) | 35.0 (10.0) | 9 (75%) | 4 (36%) | White: 11 Other: 1 | White: 7 Other: 4 | Remission or mild activity (FC < 250 μg/g and CDAI <220) - |
| Trindade et al., 2021; Portugal | ACT | CAU | 53 | IBD | 12 weeks | albumin, CRP, FC | 2 | not reported | | 13 (54%) | 16 (55%) | not reported | | - excluded diagnoses of psychiatric disorder, severe depression, suicidal ideation |
| Van Den Brink et al., 2019*; Netherlands | CBT | CAU | 35 | IBD | 12 weeks | CRP, FC | 3 | 22.19 (2.3) | 22.06 (2.3) | 16 (84%) | 10 (63%) | not reported | | - excluded diagnoses of psychiatric disorders, depression and anxiety |
| Vogelaar et al., 2011a; Netherlands | Problem Solving Therapy | CAU | 29 | CD | 12 weeks | CRP | 3 | 30.9 (8.1) | 32 (8.9) | Frequency unclearly reported (78%) | Frequency unclearly reported (65%) | not reported | | Remission (HBI < 5) Excluded anxiety and depression (≥10 HADS subscales) or psychiatric disorder diagnosis |
| Vogelaar et al., 2011b; Netherlands | Solution Focused Therapy | CAU |  | CD | 12 weeks | CRP | 3 | 29.9 (6.9) | 32 (8.9) | Frequency unclearly reported (75%) | Frequency unclearly reported (65%) | not reported | | Remission (HBI < 5) Excluded anxiety and depression (≥10 HADS subscales) or psychiatric disorder diagnosis |
| Wynne et al., 2019; Ireland | ACT | CAU | 122 | IBD | 8 weeks | leucocytes, albumin, CRP, FC, cortisol | 2 | 40.6 (11.2) | 39.9 (12.2) | 20 (54%) | 23 (55%) | not reported | | Remission or mild (CD: short CDAI ≤219, UC: Mayo ≤5) - |
| Xie et al., 2014; China | Antidepressant (Flupentixol & Melitracen) | Placebo | 80 | UC | 8 weeks | endoscopy (Baron) | 2 | whole sample: 36.5 (11.2) | | whole sample: 48 (60%) | | not reported | | Severe activity excluded (DAI 11-12) Mild-moderate anxiety/depression (HADS 8-14) included, Severe anxiety/depression (HADS 15-21) excluded |
| Zhang et al., 2020a; China | Psychoeducation | CAU | 120 | IBD | 4 weeks | IL-6, IL-17, IL-23, IL-35 | 1.5 | 37.22 (5.34) | 35.48 (4.96) | 12 (40%) | 14 (47%) | not reported | | - excluded psychiatric diagnoses |
| Zhang et al., 2020b; China | Peer support | CAU |  | IBD | 6 weeks | IL-6, IL-17, IL-23, IL-35 | 1.5 | 36.85 (4.58) | 35.48 (4.96) | 13 (43%) | 14 (47%) | not reported | | - excluded psychiatric diagnoses |
| Zhang et al., 2020c; China | Psychoeducation & Peer support | CAU |  | IBD | 6 weeks | IL-6, IL-17, IL-23, IL-35 | 1.5 | 38.46 (6.18) | 35.48 (4.96) | 12 (40%) | 14 (47%) | not reported | | - excluded psychiatric diagnoses |

* indicates that the study was conducted in both adults and children, but the table reports data from the adult sub-sample only.

ACT: Acceptance and Commitment Therapy, CAU: care as usual, CBT: Cognitive Behavioural Therapy, CD: Crohn’s disease, CDAI: Crohn’s disease activity index, CRP: C-Reactive Protein, DAI: disease activity index, DASS: Depression Anxiety and Stress Scales, ECP: Eosinophil cationic protein, ESR: erythrocyte sedimentation rate, FC: faecal calprotectin, FL: faecal lactoferrin, HADS: Hospital Anxiety and Depression Scale, HBI: Harvey Bradshaw Index, IBD: Inflammatory Bowel Disease, IBDQ: Inflammatory Bowel Disease Questionnaire, IBS: Irritable Bowel Syndrome, IgA: immunoglobulin A, IL: interleukin, MBCT: Mindfulness Based Cognitive Therapy, MBSR: mindfulness based stress reduction, PSS: Perceived Stress Scale, QoL: Quality of Life, RI: Rachmilewitz index, TNF: tumour necrosis factor, UC: ulcerative colitis, WBC: white blood cell count

# Supplementary Table 5. TIDieR table of psychosocial/behavioural intervention content.

| **Reference** | **Brief name** | **Intervention provider (level of training)** | **Modes of delivery (format)** | **Location** | **When/ how much?** | **Tailoring or adaptations** | **Intervention adherence assessed? (Strategies to enhance adherence)** | **Therapist fidelity assessed (strategies to assess fidelity)** |
| --- | --- | --- | --- | --- | --- | --- | --- | --- |
| **Berrill et al., 2014** | Mindfulness & CBT | 1 x therapist (counselling and psychotherapy qualifications) | Face-to-face, individually | University Hospital of Wales, Cardiff | 6 x 40 min sessions; 16 weeks | NR | Yes | NR |
| **Cramer et al., 2017** | Yoga | 3 x yoga instructors (a physician, a nutritional scientist, an individual trained in adult education) | Face-to-face; group | NR | 12 x 90 min; 12 weeks | Yes (tailored to physical and psychological concerns associated with UC, including reducing stress, improving QoL and bowel symptoms) | Yes | NR (instructors required to adhere to protocol) |
| **Cronin et al., 2019** | Exercise | 1 x gym instructor | Training programme face-to-face then participants exercised on their own. | Exercise facilities at Mardyke Arena at University College Cork, Ireland | 24 x 25-40 mins; 8 weeks | NR | Yes | NR (study-specific gym instructor) |
| **Elsenbruch et al., 2005** | Mind Body Therapy (Stress Management, Exercise & Diet advice, CBT) | NR | NR | NR | 6 x 60 min; 10 weeks |  | No | NR |
| **Ewais et al., 2021** | MBCT | 1 x mental health professional (teacher in MBCT, experience in chronic illness) | Face-to-face; group (8 participants) | NR | 8 x 120 mins; 8 weeks | Yes (adapted for young adults and IBD with gut-brain axis and IBD mindfulness content) | Yes | Yes (video recordings and discussion with supervisor about adherence to protocol) |
| **Gavrilescu et al., 2020** | CBT & Psychoeducation | 1 x clinical psychologist | Face-to-face; group (7-8 participants) | Tertiary Hospital | 7 X 90 mins; monthly | Yes (focussed on coping in IBD) | No | NR (supervision and trained by university) |
| **Gerbarg et al., 2015** | Breathing, Movement and Meditation | 1 x certified instructor | Face-to-face; group | Intervention: NR  Control: Jill Roberts Centre for IBD, New York Presbyterian Hospital | Intervention: 2 x 360 mins 2 days; 6 x 90 (6 weeks); 5 x 90 mins (20 weeks) weekly, then 1 x 90  Control: 1 x 360 mins; 2 x 90 mins | NR | No | NR (study-specific instructor) |
| **Gonzalez-Moret et al., 2020** | Mindfulness | 1 x mindfulness teacher (able to teach instructor courses) | Face-to-face: group Online: individually | NR | Face-to-face: 4 x 120 mins; 8 weeks Internet: 8 x 60 mins; 8 weeks | NR | Yes | NR |
| **Goren et al., 2020** | CBT & MBSR | social workers (trained in CBT & MBSR) | Online; NR (group or individual) | NR | 12 over 12 weeks (minutes NR) | NR | No | NR |
| **Goren et al., 2022** | CBT & MBSR | social workers (trained in CBT & MBSR) | Online; individually | Online | 7 X 60 mins; 12 weeks (homework 2 x 10 mins; daily). | NR | Yes (SMS reminders) | NR (videoconferences had structured protocol) |
| **Jedel et al., 2014** | MBSR | 1 x physician (15 years of MBSR experience), 1 x psychologist (MBSR experience) | NR | NR | 8 x 120-150 mins; 8 weeks | NR | Yes | NR |
| **Jedel et al., 2022** | MBSR | 1 x psychologist (trained in MBSR) | Face-to-face; group | NR | 8 x 90-120 mins; 8 weeks (1 x 4 hour optional session. Homework; weekly) | NR | Yes | Adaptions made based on group size |
| **Klare et al., 2015** | Exercise (moderate intensity) | NR | Face-to-face; group (implied) | Outside | 30 sessions; 10 weeks | NR | Yes | NR |
| **Langhorst et al., 2020** | Lifestyle modification (exercise, yoga, stress management, communication, habits, communication, home remedies, cooking classes) | physicians and instructors (trained in protocol) | Face-to-face; group | NR | 10 x 6 hour; 10 weeks | Yes (all techniques evidence-based for IBD) | Yes | NR (PI attended all sessions) |
| **Langhorst et al., 2022** | Lifestyle modification & CBT & MBSR | 1 x gastroenterologist and 1 x instructors (trained in protocol) | Face-to-face; group (5-7 participants) | Outpatient department, Department of Internal and Integrative Medicine, Sozialstiftung Bamberg, Germany | 10 x 6 hour; 10 weeks | Yes (all techniques evidence-based for IBD) | Yes | NR |
| **Liang et al., 2022** | Antidepressant (Venlafaxine) | Self-administered by participant | Tablet taken at home | Participant home | 75 mg daily first week, 150 mg daily until study end | NR | No | NR |
| **March-Lujan et al., 2021** | Guided Imagery and Music Therapy | 1 x postgraduate professional (trained in BMGIM); 1 x BMGIM specialist | Face-to-face; group (5-6 participants) | NR | 8 x 120 mins; 8 weeks | NR | No | NR (session structured to be uniform) |
| **Mikocka-Walus, et al. (2015)** | CBT | psychologist | Choice of face-to-face or online. | Online or unspecified | 10 x 120 mins; 10 weeks | Yes (designed by clinical psychologist at hospital) | Yes | NR (same protocol used for both arms) |
| **Mikocka-Walus, et al., 2017** | Antidepressant (Fluoxetine) | Self-administered by participant | Tablet taken at home | Participant home | 20 mg daily | NR | No | NR |
| **Peerani et al., 2022** | Multicomponent Stress Reduction (mindfulness, yoga, CBT, positive psychology) | Web-based programme | Online (including 1 face-to face orientation); individual | Participant home | 2-3 X 30 mins videos; 12 weeks | Yes (developed and delivered by qualified personnel, including IBD nutrition) | Yes (Weekly touchpoint with researcher) | NA – web-based programme |
| **Sharma et al., 2015** | Yoga | 1 x certified yoga trainer | Week 1: Face-to-face, Week 2-8: daily practice at home | Participant home | 60 mins daily; 8 weeks | NR | Yes (diaries, telephone support) | NR |
| **Tew et al., 2019a** | Exercise (high intensity interval training) | 1 x research assistant (trained in exercise protocol | Face-to-face; group (4 participants) | Exercise facilities at University of East London and University of Winchester | 36 x 28 mins; 12 weeks | NR | Yes | NR (specific training on intervention delivery) |
| **Tew et al., 2019b** | Exercise (moderate intensity) | 1 x research assistant (trained in exercise protocol | Face-to-face; group (4 participants) | Exercise facilities at University of East London and University of Winchester | 36 x 30 mins; 12 weeks | NR | Yes | NR (specific training on intervention delivery) |
| **Trindade et al., 2021** | ACT | certified therapists (ACT experience; MSc in Clinical Psychology) | Face-to-face; group (9-12 participants) | NR | 9 x 120 mins; 9 weeks | Yes (tailored from cancer to IBD following assessment of psychological literature) | Yes | NR (specific training and manual on intervention delivery) |
| **Van Den Brink et al., 2019** | CBT | NR | Face-to-face (6 sessions), Telephone (4 sessions) | NR | 10 x 60 min (optional 3 x 60 min); 12 weeks, 3 x booster sessions over 36 weeks | Yes (tailored to 21-25 years) | Yes | NR |
| **Vogelaar et al., 2011a** | Problem Solving Therapy | 1 x psychotherapist (experienced in PST courses) | NR | NR | 10 sessions; 12 weeks | Yes (adjusted to patient population) | Yes | NR |
| **Vogelaar et al., 2011b** | Solution Focused Therapy | 1 x psychotherapist (experienced in SFT courses) | NR | NR | 5 sessions; 12 weeks | Yes (adjusted to fatigue management in IBD) | Yes | NR |
| **Wynne et al., 2019** | ACT | 1 x psychologist (ACT trainer) | Face-to-face; group (14-16 participants) | NR | 8 x 90 mins; 8 weeks | Yes (tailored to IBD and reducing stress) | Yes | Yes (all sessions observed by independent psychologist) |
| **Xie et al., 2014** | Antidepressant (Flupentixol & Melitracen) | Self-administered by participant | Tablet taken at home | Participant home | 5 mg of flupentixol, 10 mg of tetramethylthracene; twice daily | NR | No | NR |
| **Zhang et al., 2020a** | Narrative medicine-based health education | doctors | NR; individually | NR | 1 x 60 mins, 3 additional sessions; 4 weeks | NR | NR | NR |
| **Zhang et al., 2020b** | Support group | doctors, nurses, psychotherapists, and nutritionists | Online; group | Online | flexible; 6 weeks | NR | NR | NR |
| **Zhang et al., 2020c** | Narrative medicine-based health education (NMBH)  and  Support Group (SG) | NMBH: doctors  SG: doctors, nurses, psychotherapists, and nutritionists | NMBH: NR; individually  SG: Online; group | NMBH: NR  SG: Online | NMBH: 1 x 60 mins, 3 additional sessions; 4 weeks  SG: flexible; 6 weeks | NR | NR | NR |

ACT: Acceptance and Commitment Therapy, BMGIM: Bonny Method of Guided Imagery and Music, CBT: Cognitive Behavioural Therapy, IBD: Inflammatory Bowel Disease, MBCT: Mindfulness Based Cognitive Therapy, MBSR: Mindfulness-based stress reduction, NMBH: Narrative medicine-based health education NR: not reported, PI: Principal Investigator, PST: problem solving therapy, SFT: solution focussed therapy, SG: Support group, SMS: short message service

# Supplementary Figure 1. Scatter plot of Study Effect Size and Study Average Age

# Supplementary Figure 2. Scatter plot of Study Effect Size and Study Gender Proportion

# Supplementary Figure 3. Funnel plot to assess publication bias for studies investigating the effect psychosocial/behavioural interventions on faecal calprotectin. A scatterplot of treatment effect against a measure of study precision, to visually inspect the studies for publication bias and systematic heterogeneity.

# Supplementary Figure 4. Forest plot of leave-one-out sensitivity analysis for faecal calprotectin

# Supplementary Figure 5. Funnel plot to assess publication bias for studies investigating the effect psychosocial/behavioural interventions on C-Reactive Protein. A scatterplot of treatment effect against a measure of study precision, to visually inspect the studies for publication bias and systematic heterogeneity.

# Supplementary Figure 6. Forest plot of leave-one-out sensitivity analysis for C-Reactive Protein.

# Supplementary Table 6. Risk of Bias of Included Studies

| **Reference** | Risk of bias arising from the randomization process | Risk of bias due to deviations from assignment to intervention (blinding) | Risk of bias due to missing outcome data | Risk of bias in measurement of outcome | Risk of bias in selection of the reported result | **Overall risk** |
| --- | --- | --- | --- | --- | --- | --- |
| Berrill et al., 2014 | low risk | low risk | some concerns | low risk | low risk | some concerns |
| Cramer et al., 2017 | low risk | low risk | low risk | low risk | high risk | high risk |
| Cronin et al., 2019 | low risk | high risk | some concerns | low risk | low risk | high risk |
| Elsenbruch et al., 2005 | some concerns | high risk | low risk | low risk | high risk | high risk |
| Ewais et al., 2021 | low risk | low risk | some concerns | low risk | low risk | some concerns |
| Gavrilescu et al., 2020 | some concerns | high risk | low risk | low risk | high risk | high risk |
| Gerbarg et al., 2015 | high risk | low risk | high risk | low risk | some concerns | high risk |
| Gonzalez-Moret et al., 2020 | low risk | low risk | low risk | low risk | high risk | some concerns |
| Goren et al., 2020* | low risk | some concerns | some concerns | low risk | high risk | some concerns |
| Goren et al., 2022 | high risk | high risk | some concerns | low risk | some concerns | high risk |
| Jedel et al., 2014 | low risk | low risk | high risk | low risk | some concerns | high risk |
| Jedel et al., 2022 | low risk | some concerns | low risk | low risk | low risk | some concerns |
| Klare et al., 2015 | low risk | high risk | some concerns | low risk | high risk | high risk |
| Langhorst et al., 2020 | low risk | low risk | low risk | low risk | low risk | low risk |
| Langhorst et al., 2022 | low risk | low risk | some concerns | low risk | low risk | some concerns |
| Liang et al., 2022 | low risk | low risk | low risk | low risk | some concerns | some concerns |
| March-Lujan et al., 2021 | low risk | low risk | low risk | low risk | some concerns | some concerns |
| Mikocka-Walus, et al. 2015 | low risk | low risk | low risk | low risk | high risk | high risk |
| Mikocka-Walus, et al. 2017 | low risk | low risk | low risk | low risk | low risk | low risk |
| Peerani et al., 2022 | low risk | some concerns | high risk | low risk | high risk | high risk |
| Sharma et al., 2015 | low risk | low risk | some concerns | low risk | high risk | high risk |
| Tew et al., 2019 | low risk | low risk | low risk | low risk | high risk | high risk |
| Trindade et al. 2021 | low risk | low risk | low risk | low risk | low risk | low risk |
| Van Den Brink et al., 2019 | low risk | low risk | low risk | low risk | low risk | low risk |
| Vogelaar et al., 2011 | low risk | some concerns | some concerns | low risk | high risk | some concerns |
| Wynne et al., 2019 | low risk | low risk | low risk | low risk | high risk | some concerns |
| Xie et al., 2014 | some concerns | low risk | low risk | low risk | high risk | some concerns |
| Zhang et al., 2020 | high risk | high risk | low risk | low risk | high risk | high risk |

* indicates potential for risk of bias judgment to be excessively harsh, as only an abstract was published
